# Comprehensive sampling of mitochondrial genomes substantiates the Neoproterozoic origin of land plants

**DOI:** 10.1016/j.xplc.2025.101497

**Published:** 2025-09-05

**Authors:** Shuai-Ya Hu, Gongle Shi, Cheng-Ao Yang, Yves Van de Peer, Zhen Li, Jia-Yu Xue

**Affiliations:** 1 College of Horticulture, Bioinformatics Center, Academy for Advanced Interdisciplinary Studies, Nanjing Agricultural University, Nanjing 210095, China; 2 Department of Plant Biotechnology and Bioinformatics, Ghent University, 9052 Ghent, Belgium; 3 VIB-UGent Center for Plant Systems Biology, 9052 Ghent, Belgium; 4 State Key Laboratory of Palaeobiology and Stratigraphy, Nanjing Institute of Geology and Palaeontology, Chinese Academy of Sciences, Nanjing, China; 5 Department of Biochemistry, Genetics and Microbiology, University of Pretoria, Pretoria 0028, South Africa

**Keywords:** mitochondrial genomes, phylogenomics, molecular models, fossil calibration, rate heterogeneity

## Abstract

Molecular phylogenetics elucidates the evolution and divergence of green plants by analyzing sequence data from diverse sources. Notably, phylogenetic reconstruction based on mitochondrial genes often shows incongruence with results from nuclear and chloroplast genes. Although the uniparental inheritance and conservatively retained protein-coding genes of mitochondrial genomes inherently exclude certain confounding factors that affect phylogenetic reconstruction—such as hybridization and gene loss—the use of mitochondrial genomes for phylogeny and divergence-time estimation has remained limited. Here, we assembled a comprehensive dataset of 565 mitochondrial genomes representing all major lineages of green plants. Applying multiple partitions and phylogenetic models, our mitochondrial-based phylogenies support paraphyly in both bryophytes and charophytes, place hornworts (Anthocerotaceae) as sister to all tracheophytes, and recover stoneworts (Charophyceae) as sister to land plants. We systematically evaluated the influence of factors in mitochondrial coding sequences, including GC-content heterogeneity and codon-usage bias. Furthermore, by rigorously testing seven dating strategies, we assessed the impact of confounding elements affecting divergence-time estimates, such as fossil calibration number and prior settings, as well as rate heterogeneity among sites and across lineages. Our dating analyses support a Neoproterozoic origin (crown age) of land plants and a Triassic origin of angiosperms, consistent with nuclear evidence. In conclusion, we emphasize the importance of exploring alternative partitioning strategies and addressing among-lineage heterogeneity in both phylogenetic and dating analyses, with extended sampling and careful data pruning to minimize systematic error in phylogenetic inference.

## Teaser

This study presents phylogenetic analyses based on565 mitochondrial genomes spanning all major green plant lineages, identifying hornworts as sister to tracheophytes and Charophyceae as sister to land plants, while revealing paraphyly in both bryophytes and charophytes. By testing multiple partitioning schemes, models, and seven dating strategies—while adjusting for GC content, codon-usage bias, and rate heterogeneity—the study demonstrates the importance of careful modeling and data pruning to reduce systematic error. The dating analyses support a Neoproterozoic crown age for land plants and a Triassic origin of angiosperms, consistent with nuclear evidence.

## Introduction

Embryophytes (land plants), the most diverse plant lineage in terrestrial habitats, together with charophytes and chlorophytes (green algae), form the group known as Viridiplantae, or green plants. Understanding the evolutionary history of green plants, particularly land plants, provides critical insights into the formation of Earth’s modern ecosystems and establishes a theoretical framework for contemporary biological and ecological research. Green algae have substantially influenced Earth’s ecosystems since their ancestors engaged in interactions with fungi or prokaryotes in aquatic and terrestrial environments at least 1000 million years ago (Ma) (Knauth and Kennedy, 2009; Strother et al., 2011; Lutzoni et al., 2018; Del Cortona et al., 2020; McCourt et al., 2023). The diversity of terrestrial green algae (Lewis and Flechtner, 2002; Gray et al., 2007; Holzinger and Karsten, 2013; Fučíková et al., 2014; Mikhailyuk et al., 2018; Del Cortona et al., 2020) and their interactions with soil microbes established the foundation for the subsequent evolution of land plants (McCourt et al., 2023). Subsequent co-evolution among land plants, animals, and fungi has profoundly shaped the modern Earth. Mapping traits onto the phylogeny of green plants allows a clearer understanding of the origins of key features—such as multicellularity, terrestrial adaptation, life cycles, anatomical structures, and stress-resistance physiology—that enabled colonization of land (Qiu et al., 2012). Addressing these questions depends on robust phylogenetic inference, comprehensive fossil evidence, and precise estimates of divergence time.

Correspondingly, controversies persist concerning the phylogenetic relationships and divergence times of the major clades within green plants. The incongruences include, but are not limited to, (1) which member of the ZCC clade—Zygnematophyceae, Coleochaetophyceae, or Charophyceae—is the closest relative of Embryophyta; (2) whether bryophytes (i.e., Marchantiophyta, Bryophyta, Anthocerotaceae, or mosses, liverworts, and hornworts) are paraphyletic or monophyletic and which, if any, represent the earliest diverging lineage of Embryophyta and the closest relative of Tracheophyta; and (3) the relationships among Ginkgoopsida, Cycadopsida, Pinopsida, and Gnetopsida, as well as the origin and early diversification of Angiospermae (Cox, 2018; Puttick et al., 2018; Qiu and Mishler, 2024). Numerous phylogenies have been proposed, yet consensus remains elusive. A widely held view has been a stepwise increase in organismal complexity, from unicellular and simple filamentous forms such as extant *Zygnema*, to pseudoparenchymatous apical cell-bearing *Coleochaete*, and ultimately to complex multicellular forms with rhizoids and stem-like structures such as *Chara*. This hypothesis has been supported by many studies (Lewis and McCourt, 2004; Qiu et al., 2006, 2012; Qiu, 2008; Becker and Marin, 2009; Turmel et al., 2013; Liu et al., 2014). Recently, however, studies leveraging large-scale nuclear genome datasets have increasingly supported Zygnematophyceae as the sister group of land plants, introducing additional phylogenetic uncertainty (Civáň et al., 2014; Cox et al., 2014; Ruhfel et al., 2014; Wickett et al., 2014; Zhong et al., 2014; Lemieux et al., 2016; Puttick et al., 2018; Cheng et al., 2019; One Thousand Plant Transcriptomes Initiative, 2019; Del Cortona et al., 2020; Su et al., 2021). Another phylogenetic “problem” concerns the monophyletic origin of bryophytes, which has gained overwhelming support from recent nuclear genome data (Wickett et al., 2014; Gitzendanner et al., 2018; Puttick et al., 2018; One Thousand Plant Transcriptomes Initiative, 2019; Sousa et al., 2019; Harris et al., 2020; Zhang et al., 2020; Su et al., 2021; Harris et al., 2022; Yang et al., 2022). This view contrasts with the more traditional hypothesis of bryophyte paraphyly, where hornworts are sister to tracheophytes (Qiu, 2008; Turmel et al., 2013; Liu et al., 2014; Ruhfel et al., 2014). Such phylogenetic uncertainties impede resolution of two fundamental questions in embryophyte evolution: are key traits the result of adaptation or exaptation (preadaptation) (de Vries and Archibald, 2018), and do they reflect antithetic or homologous alternations (Königlich-böhmische Gesellschaft der Wissenschaften, 1874; Qiu et al., 2012)? Accurate timescale estimates derived from stably resolved phylogenies are essential to decide the timing of green plant body plan assembly and its influence on global biogeochemical cycles (Donoghue et al., 2021). The scarcity and uncertain interpretation of fossils from early green and land plants lead to substantial discrepancies in evolutionary timescale estimates, even when molecular data are analyzed with advanced calibration strategies and refined molecular models (Blank, 2013; Leliaert et al., 2016; Yang et al., 2016; Morris et al., 2018; Nie et al., 2020; Su et al., 2021; Harris et al., 2022).

Mitochondrial molecular data have long served as phylogenetic markers for testing diverse evolutionary hypotheses (Beckert et al., 1999; Qiu et al., 1999a, 1999b, 2010; Bowe et al., 2000; Forrest et al., 2006; Mennes et al., 2013). In the genomic era, however, relatively few studies have utilized complete mitochondrial genomes as sources of sequence data for phylogenetic reconstruction, particularly within frameworks requiring elaborate model-fitting approaches. These approaches include accounting for variability sorting, codon degeneracy, nucleotide (nt) data with non-stationary composition models, and amino acid (aa) data with non-stationary composition models (Liu et al., 2014; Sousa et al., 2020; Xue et al., 2022). Comparatively, mitochondrial data provide a clearer signal of genetic relationships due to uniparental inheritance and thus yield more straightforward monophyletic relationships of gene families than nuclear data, where complicating factors such as hybridization, gene duplication, and gene loss are present. These clearer signals enable researchers to focus more directly on the role of systematic error in phylogenetic reconstruction. Although mitochondrial gene sequences are the most conserved among mitochondrial, plastid, and nuclear genomes (Palmer and Herbon, 1988a; Knoop, 2004; Xue et al., 2022), challenges to tree inference remain. These include substitutional saturation, GC-content heterogeneity, and codon-usage bias, which are particularly problematic at deeper evolutionary scales (Liu et al., 2014). Here, we implemented extensive sampling of more than 500 species and applied multiple phylogenetic methods for both tree reconstruction and molecular dating, to propose a refined timescale for green plant evolution based on mitochondrial genomes.

## Results

### Supermatrix construction and characteristics

To reconstruct a phylogeny encompassing all major clades of green plants, we assembled a supermatrix by concatenating nt sequences of 39 mitochondrial protein-coding genes from 565 species representing 129 orders across Viridiplantae, with eight Rhodophyta species as outgroup (Supplemental Table 1). After multiple sequence alignment and gap trimming, the final matrix contained 27 114 sites, of which 21 947 were parsimony informative: 7 045 at first, 6 351 at second, and 8 551 at third codon positions.

**Figure 1:**
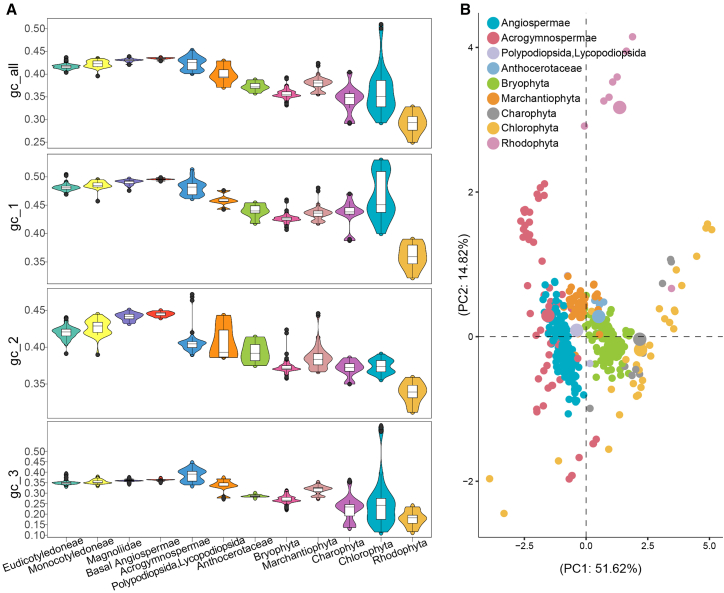
Data characteristics of the concatenated nucleotide matrix from 39 mitochondrial genes across 565 plants. **(A)** Violin plots illustrating GC-content differences, with the *x* axis representing 11 major lineages of Viridiplantae and the outgroup Rhodophyta. Each lineage is indicated by a distinct color. **(B)** PCA plot showing codon-usage bias, with each dot representing a species and colors distinguishing the lineages.

Because the dataset was densely sampled and provided broad lineage coverage, it enabled detection of molecular patterns, namely the defining characteristics of key lineages and the degrees of divergence among them. These characteristics were considered representative and were subsequently incorporated as impact factors into our phylogenetic models and dating strategies. As an initial step, we compared GC content across major Viridiplantae lineages: Eudicotyledoneae, Monocotyledoneae, Magnoliidae, basal Angiospermae, Acrogymnospermae, Polypodiopsida plus Lycopodiopsida (i.e., ferns and lycophytes), Anthocerotaceae, Bryophyta, Marchantiophyta, Charophyta, and Chlorophyta. Overall, Tracheophyta (Angiospermae, Acrogymnospermae, Polypodiopsida, and Lycopodiopsida) displayed the highest GC content, bryophytes (Anthocerotaceae, Bryophyta, and Marchantiophyta) showed intermediate values, and Charophyta/Chlorophyta exhibited the lowest content. The distribution of GC content within Chlorophyta was highly dispersed. Across all groups, the third codon positions showed the lowest GC content, while the first codon positions displayed the highest (Figure 1A and Supplemental Table 2). Notably, for the third codon position, three lineages—Chlorophyta, Bryophyta, and Anthocerotaceae—exhibited strikingly similar mean GC content values of 0.27, 0.27, and 0.29, respectively. In contrast, Charophyta showed a mean value of 0.22 whereas Tracheophyta displayed 0.36.

Principal-component analysis (PCA) of relative synonymous codon usage (RSCU) values across all key lineages revealed distinct codon usage patterns among Tracheophyta, Bryophyta, Charophyta plus Chlorophyta, and the outgroup Rhodophyta (red algae) (Figure 1B and Supplemental Table 3). Our results indicated that principal component 1 (PC1) clearly separates Tracheophyta from Charophyta and Chlorophyta. Principal component 2 (PC2) further suggests that codon usage differences are most apparent between Viridiplantae and Rhodophyta. Notably, Acrogymnospermae and Chlorophyta display the most divergent codon usage patterns compared with other Viridiplantae lineages. Within bryophytes, Bryophyta and Marchantiophyta can be distinguished by both PC1 and PC2, reflecting a strong codon usage bias, with Bryophyta resembling algae and Marchantiophyta more closely resembling Tracheophyta (Figure 1B).

To better illustrate the early branches of the green lineage, we constructed a reduced dataset of 65 species that included Glaucocystophyceae, representative taxa from ten major lineages, *Phlegmariurus squarrosus*, and six additional Glaucocystophyceae species (Supplemental Table 4). GC content analysis of this dataset also revealed differences among the eight major lineages. In the PCA, PC1 (*x* axis) divide the eight lineages into two groups, with all Embryophyta clustering on the left and all algal lineages clustering on the right, while the Rhodophyta branch was distinctly separated along PC2 (*y* axis). The Glaucocystophyceae branch overlapped with Chlorophyta and streptophyte algae in both PC1 and PC2, consistent with recent studies that classified Glaucocystophyceae as the closest sister group of Viridiplantae (One Thousand Plant Transcriptomes Initiative, 2019) (Supplemental Figure 2A). In GC-content analyses, a clear trend emerged: Tracheophyta exhibited the highest GC content in all four datasets, with mean values of 0.427, 0.485, 0.438, and 0.358 for GC_all (all codon positions), GC_1 (first codon position), GC_2 (second codon position), and GC_3 (third codon position), respectively (Supplemental Table 5). The GC content of all other green plant lineages (Anthocerotophyta, Bryophyta, Marchantiophyta, streptophyte algae, and Chlorophyta) was broadly similar and slightly lower than that of Tracheophyta, while Glaucocystophyceae and Rhodophyta exhibited substantially lower GC content compared with Viridiplantae lineages (Supplemental Figure 2B).

### Phylogenetic analyses

We evaluated phylogenetic stability using maximum-likelihood (ML) and Bayesian inference (BI) across three data types customized to our dataset, then compared conflicting signals between the phylogeny generated here and that from the 1000 Plants (1KP) project.

To account for among-site heterogeneity, we employed a partition model under the ML framework. To examine phylogenetic consistency, we performed ML analyses on three datasets—nt (all codon positions: first, second, third), nt12 (first and second codon positions only), and aa (amino acid sequences), each capturing different types of evolutionary information. The nt dataset contains both synonymous and non-synonymous substitutions; however, the third codon position evolves rapidly, accumulates substitutional saturation, and may introduce misleading signals. The nt12 dataset excludes all third-codon positions, producing more conserved sequences but at the cost of losing a substantial portion of sequence variation and informative sites. The aa dataset reflects only non-synonymous substitutions (i.e., those altering protein sequences) and is therefore strongly influenced by functional constraints. Because aa sequences represent only one-third the length of nt sequences, they also lack a large proportion of informative sites, potentially limiting resolution for more recent divergences. Nevertheless, the aa dataset, with 20 possible character states per site compared to four in nt data, exhibits very low substitutional saturation and is thus particularly suitable for resolving deeper phylogenies.

We next performed a homogeneous BI analysis to validate the phylogeny inferred from the ML method. BI integrates prior beliefs with likelihood to estimate posterior probabilities (PPs) and employs Markov Chain Monte Carlo (MCMC) to sample trees from the posterior distribution, thereby incorporating phylogenetic uncertainty into its results. The homogeneous model assumes that mutation rates across all sites follow a single statistical distribution, comparable to the setting used in the ML. The term “homogeneous” is utilized here to distinguish it from the “heterogeneous” model employed in an alternative BI analysis. The most evident conclusion drawn from the “supermatrix characteristics” analyses above is the presence of among-lineage compositional heterogeneity, largely due to GC-content variation and codon-usage bias (Figure 1). To adjust for this factor, we implemented a heterogeneous model in another BI analysis using the Python library P4. Rhodophyta was consistently selected as the outgroup in all analyses.

### Phylogenetic results of six different partition schemes

The third codon position often evolves rapidly and exhibits the lowest GC content across lineages, reflecting compositional heterogeneity (Figure 1A). To evaluate the effects of among-site and among-gene heterogeneity, we constructed six subsets with different partition schemes from the concatenated nt dataset, categorized by genes and codon positions (Supplemental Table 6) (see Methods). We applied a partition model implemented in RAxML, allowing model parameters to be estimated for each partition. All six schemes yielded identical backbone topologies for the nine major green plant lineages—Chlorophyta, Charophyta, Marchantiophyta, Bryophyta, Anthocerotaceae, Lycopodiopsida, Polypodiopsida, Acrogymnospermae, and Angiospermae—with comparable bootstrap support (Supplemental Figures 4 and 6–8). Therefore, increasing the number of partitions (from one to 117) did not lead to any substantial changes in the final topology or bootstrap support of the backbone phylogeny.

### Phylogenetic results of three datasets

**Figure 2:**
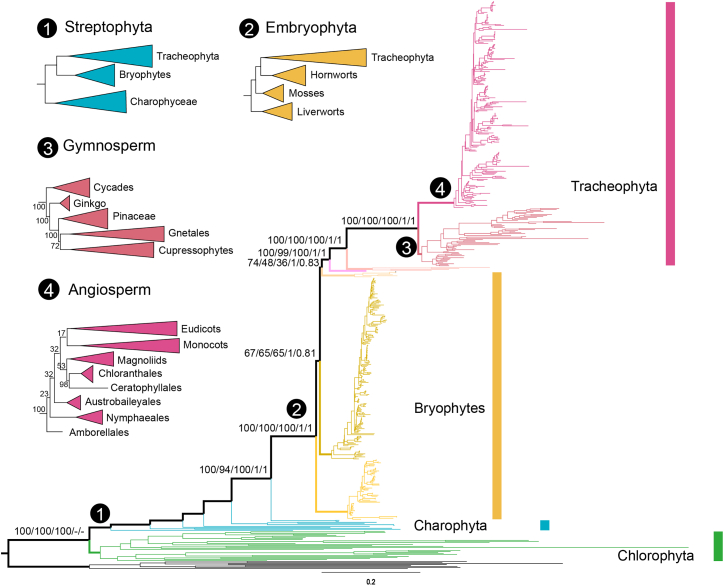
“Backbone” phylogenetic tree inferred from nucleotide sequences using all three codon positions. Numbers on eight internal nodes represent bootstrap values (BS) or posterior probabilities (PP) for topologies of eight backbone lineages, inferred from three datasets (nucleotide, amino acid, and nucleotides at positions 1 and 2) and two Bayesian models (homogeneous and heterogeneous). Incongruent topologies between ML and Bayesian analyses are marked as “–.” Phylogenies of Streptophyta, Embryophyta, Acrogymnospermae, and Angiospermae are shown in the top left, corresponding to four circled numbers at each node.

The ML phylogenies inferred from the three datasets produced congruent topologies in which eight major lineages were consistently depicted as monophyletic groups: Chlorophyta, Marchantiophyta, Bryophyta, Anthocerotaceae, Lycopodiopsida, Polypodiopsida, Acrogymnospermae, and Angiospermae. Charophyta, however, was not considered monophyletic (Figure 2; Supplemental Figures 6A and 9). Chlorophyta was identified as monophyletic and as the sister lineage to all Streptophyta, with maximal support (BS_nt_ = 100%, BS_aa_ = 100%, BS_nt12_ = 100%). ML analyses of nt, aa, and nt12 datasets consistently placed Charophyceae as the sister group to all Embryophyta with strong support (BS_nt_/BS_aa_/BS_nt12_ = 100%), contradicting recent studies based on nuclear data (One Thousand Plant Transcriptomes Initiative, 2019) (marked node 1 in Figure 2). Furthermore, all three datasets indicated a paraphyletic relationship among the three bryophyte lineages, with Marchantiophyta inferred as the earliest diverging lineage of Embryophyta (BS_nt_ = 100%, BS_aa_ = 100%, BS_nt12_ = 100%) and Anthocerotaceae as sister to Tracheophyta (BS_nt_ = 74%, BS_aa_ = 48%, BS_nt12_ = 38%), although with relatively low bootstrap support (marked node 2 in Figure 2). This result contradicts several recent studies but aligns with previous research based on 60 mitochondrial genomes (Liu et al., 2014). Lycopodiopsida and Polypodiopsida were consistently resolved as successive sister groups to the remaining Tracheophyta, while Acrogymnospermae was recovered as sister to Angiospermae, with all nodes supported by bootstrap values ≥99%. In Acrogymnospermae, Cycads were inferred as the sister group of all other Acrogymnospermaes, and Pinaceae was grouped with the lineage comprising Gnetales and Cupressophytes (the “gnecup” clade) (marked node 3 in Figure 2). Among Angiospermae, Amborellales was resolved as sister to all other lineages, while Ceratophyllales and Chloranthales formed a monophyletic group that was recovered as sister to Magnoliidae; together, these were resolved as sister to a clade comprising Monocotyledoneae and Eudicotyledoneae (marked node 4 in Figure 2).

Using the aforementioned 65-species dataset, we included glaucophyte data (Glaucocystophyceae) in an additional phylogenetic analysis. ML tree inference based on both nt and aa data confirmed Glaucocystophyceae as the sister group of Viridiplantae. The three bryophyte lineages were again resolved as paraphyletic, and Charophyceae remained the closest Charophyta relatives of Embryophyta (Supplemental Figure 3).

### Phylogenetic results from Bayesian methods

To assess the reliability of the ML phylogenetic results, we conducted two Bayesian analyses using homogeneous and heterogeneous models. For the homogeneous analysis, we applied nt12–3 partition data, treating the first two codon positions as one partition and the third codon position as another, thereby considering differences in evolutionary rates between positions within a codon. In contrast, the heterogeneous Bayesian model incorporated parameters allowing rate variation among species and lineages. The homogeneous Bayesian analysis yielded a topology consistent with the ML results for the backbone relationships among the eight principal green plant lineages (Charophyta, Marchantiophyta, Bryophyta, Anthocerotaceae, Lycopodiopsida, Polypodiopsida, Acrogymnospermae, and Angiospermae), with all nodes supported at PP = 1. However, Chlorophyta was inferred as paraphyletic with relatively low support (PP = 0.86) (Figure 2 and Supplemental Figure 10A). This incongruence may reflect high GC-content heterogeneity and codon-usage bias within Chlorophyta (Figure 1), such as the use of non-canonical genetic codes (Nedelcu et al., 2000; Li et al., 2021), inter-lineage diversity in evolutionary trajectories (Nedelcu et al., 2000), reduced numbers of protein-coding genes in Chlamydomonadalean lineages (Del Vasto et al., 2015), or limitations of MCMC sampling. In the latter case, runs were typically terminated when the average standard deviation of split frequencies (ASDSF) reached <5% (ideally <1%), in consideration of computational efficiency; our focus was recovery of accurate backbone topology. Among-lineage compositional bias has long been regarded as one of the most critical systematic errors influencing phylogenetic inference. The Python package P4 performs non-stationary composition MCMC, allowing compositional variation across the tree. The results of the heterogeneous Bayesian analysis resembled those from the homogeneous Bayes analysis: (1) paraphyly of chlorophytes with low PP support for internal nodes; and (2) an identical backbone topology for eight major nodes but with different PP values—BS_Charophyceae__−__Embryophyta_ = 1, BS_Marchantiophyta__−__Stomatophyta_ = 1, BS_Bryophyta__−__(Anthocerotaceae +_ _Tracheophyta)_ = 0.81, BS_Anthocerotaceae__−__Tracheophyta_ = 0.83, BS_Lycopodiopsida__−__(Polypodiopsida + Spermatophyta)_ = 1, BS_Polypodiopsida__−__Spermatophyta_ = 1, and BS_Acrogymnospermae__−__Angiospermae_ = 1 (Figure 2 and Supplemental Figure 10B). These results indicate that compositional heterogeneity across the three bryophyte lineages exerts a measurable impact on their phylogenetic placement.

Comparison with the phylogeny from the 1KP project, which adopted nuclear genes (transcriptomes) from 1000 plant species to build a large-scale phylogenetic tree, revealed incongruence in the placement of several major lineages. Within seed plants, the position of Vitales and its relationships within Mesangiospermae and Acrogymnospermae are persistently unresolved issues (Zhang et al., 2016, 2025; Li et al., 2019; Stull et al., 2021; Hu et al., 2023; Zuntini et al., 2024). In this study, we mainly focused on two deeper phylogenetic questions: the monophyly versus paraphyly of bryophytes and the identity of the closest Charophyta relatives of Embryophyta. Most studies using nuclear data have supported bryophyte monophyly and placed Zygnematophyceae as sister to all Embryophyta (Wickett et al., 2014; Gitzendanner et al., 2018; Puttick et al., 2018; One Thousand Plant Transcriptomes Initiative, 2019; Sousa et al., 2019; Harris et al., 2020; Zhang et al., 2020; Su et al., 2021; Harris et al., 2022; Yang et al., 2022) (Supplemental Figure 5B). However, mitochondrial data have not fully resolved whether Zygnematophyceae or Charophyceae are sister to all Embryophyta, and paraphyly of bryophytes has received stronger support in other mitochondrial studies (Turmel et al., 2013; Liu et al., 2014; Sousa et al., 2020) (Supplemental Figure 5A). To further evaluate these alternatives, we conducted topology tests using IQ-TREE 2. The AU test, the KH test, and the indices bp-RELL and c-ELW all significantly rejected the hypotheses of bryophyte monophyly and Zygnematophyceae as sister to Embryophyta (Supplemental Table 7).

### Divergence-time estimation

We aimed to recalculate a credible timescale for the Viridiplantae lineage using Rhodophyta as outgroup; our main focus was resolving the divergence time of early Embryophyta, given recent debates concerning conflicting estimates of the crown age of Embryophyta (Morris et al., 2018; Su et al., 2021; Harris et al., 2022). Two datasets—designated main and accessory—were used to evaluate the accuracy and precision of our dating analyses. For the main dataset, we applied 22 fossil calibrations in strategy A, primarily following those employed by Morris et al. (2018). In strategy B, we expanded the calibrations to 55 by including 30 additional fossils with the original 22 and supplementing three algal fossil calibrations by Nie et al. (2020).

Seven (sub)strategies (see Methods, Table 1, and Supplemental Table 8) were devised to test the influence of key factors. The first three, all within strategy A, adopted 22 unequivocal fossil calibrations placed at corresponding outermost nodes at the phylum level, but with differing maximum and minimum age constraints. In strategy A1, we conservatively applied a maximum age bound of 515.5 Ma for early Embryophyta nodes based on the oldest cryptospore fossil, as in Morris et al. (2018). In strategy A2, the maximum bound for early Embryophyta nodes was extended to 1042 Ma, based on the Diabaig Formation, which represents the earliest environment suitable for land plants. In both strategies A1 and A2, we adopted 469 Ma as the minimum bound for crown Viridiplantae, considering the oldest tetrahedral tetrad fossil assigned to the Dapingian Stage of the Middle Ordovician. In contrast, strategy A3 used a newly described macrofossil, *Proterocladus antiquus*, from the Nanfeng Formation dated at 940.4 Ma, as the minimum bound for crown Viridiplantae (see Supplemental Information for detailed justification and fossil sources mentioned above, as well as discussion of controversies between the two maximum bounds and two minimum bounds). Contrasting strategy A1 with A2 allowed us to examine the impact of alternative maximum bounds on the estimated origin time (crown age) of Embryophyta, while contrasting strategy A2 with A3 assessed the effect of using different minimum bounds for crown Viridiplantae.

**Table 1:** Details of seven calibration strategies.

	Strategy A			Strategy B			
	A1	A2	A3	B1	B2	B3	B4
Fossil numbers	22	22	22	55	52	55	55
Max. bound of early Embryophyta (Ma)	515	1042	1042	1042	1042	1042	1042
Min. bound of crown Viridiplantae (Ma)	469	469	940.4	940.4	940.4	940.4	940.4
Data partitions	1	1	1	1	1	3	1
Rate-drift parameter (*σ*^2^)	(1,10)	(1,10)	(1,10)	(1,10)	(1,10)	(1,10)	(10,10)

The rate-drift parameter (*σ*^2^) specifies variability in rates across clades.

Because we observed a clear improvement in model fit when relaxing the maximum age bound for early Embryophyta calibrations from 515 Ma to 1042 Ma in strategy A2, we considered the estimated timescale based on the latter bound to be more credible. Moreover, the use of an older fossil to calibrate the crown Viridiplantae node (940.4 Ma) did not alter the estimated timescale in strategy A3 (described below). Therefore, across all four substrategies of strategy B, we retained 1042 Ma as the maximum bound for early Embryophyta and 940.4 Ma as the minimum bound for crown Viridiplantae. In strategy B1, we expanded the 22 fossil calibrations used in strategy A3 by adding 33 “low-level” fossils (e.g., at crown nodes of Classes, Orders, Families, or Genera), yielding 55 calibrations. In strategy B2, we excluded three uncertain algal fossils, leaving 52 calibrations. Thus, contrasting strategies A3, B1, and B2 allowed evaluation of the effect of fossil calibration number. Furthermore, given that compositional heterogeneity was observed in our main dataset (Figure 1), strategy B3 divided the molecular data into three partitions, one for each codon position, to accommodate among-site rate heterogeneity. In strategy B4, we increased rate variation across clades by adjusting the prior parameter σ^2^, thus incorporating among-lineage heterogeneity. Consequently, comparing B1 with B3 assessed the effects of data partitioning (among-site heterogeneity), while comparing B1 with B4 tested the effects of among-lineage heterogeneity (Table 1). For the accessory dataset, a subtree of 207 species preserving Order-level diversity was used to perform a clock-model test and sensitivity analysis, considering computational efficiency.

### Influence of maximum bound on divergence-time estimation

**Figure 3:**
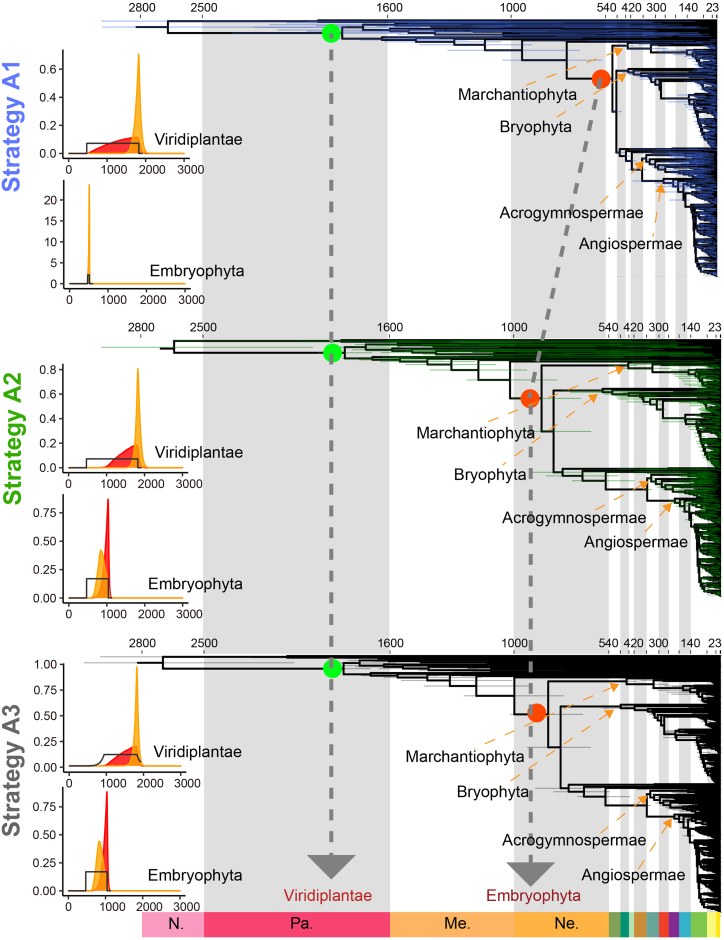
Time trees for early green plants and early Embryophyta inferred under three calibration strategies (A1–A3). Time trees for early green plants (green dots) and early Embryophyta (red dots) inferred from three different calibration strategies are shown, with corresponding time distributions on the left: fossil priors (black lines; all strategies and nodes use soft maximum and hard minimum bounds, except crown Viridiplantae in strategy A3, which uses a soft minimum bound), effective priors (orange), and posterior times (red).

The role of fossil calibrations in molecular clock studies, particularly maximum age calibrations, is subject to controversy (Morris et al., 2018; Su et al., 2021; Harris et al., 2022). Considering the recent controversy regarding the inferred crown age of Embryophyta (late Cambrian vs. Neoproterozoic) and the selection of the crown Viridiplantae minimum bound based on two fossils (Morris et al., 2018; Harris et al., 2022), we specified three strategies to test divergence times for these two critical nodes using our mitochondrial dataset with the most extensive species sampling to date and the fixed tree inferred from mitochondrial data (Figure 3). Strategy A1 (blue) set a relatively young maximum crown age (515.5 Ma) for all major Embryophyta lineages, leading to an inferred origin of early Embryophyta at ∼525 Ma (late Cambrian). Strategy A2 (green) extended the soft maximum bound of crown Embryophyta from 515.5 Ma to 1042 Ma (Su et al., 2021), resulting in a substantially older inferred origin at ∼875 Ma (Neoproterozoic). Strategy A3 examined the impact of altering the crown Viridiplantae minimum bound from 469 Ma to 940 Ma (Morris et al., 2018; Harris et al., 2022); however, no discernible difference was observed when compared with strategy A2 (Figure 3, right). In strategy A1, the effective prior and posterior distributions for the Embryophyta node nearly overlapped, particularly at their peaks, whereas in strategy A2 they considerably diverged (Figure 3, left; Supplemental Figures 11 and 12). In strategy A3, the effective prior and posterior distributions did not show any change compared with those of strategy A2 (Figure 3, left; Supplemental Figures 12 and 13). Therefore, a relaxed soft maximum calibration bound exerts considerable influence on divergence-time estimation. Given the uncertainties of the fossil record, caution is required to avoid both a priori and a posteriori overfitting. Our findings indicate that strategy A1 was primarily driven by fossil calibration constraints rather than molecular evidence.

### Influence of fossil number, data partitioning, and among-branch rate variation

**Figure 4:**
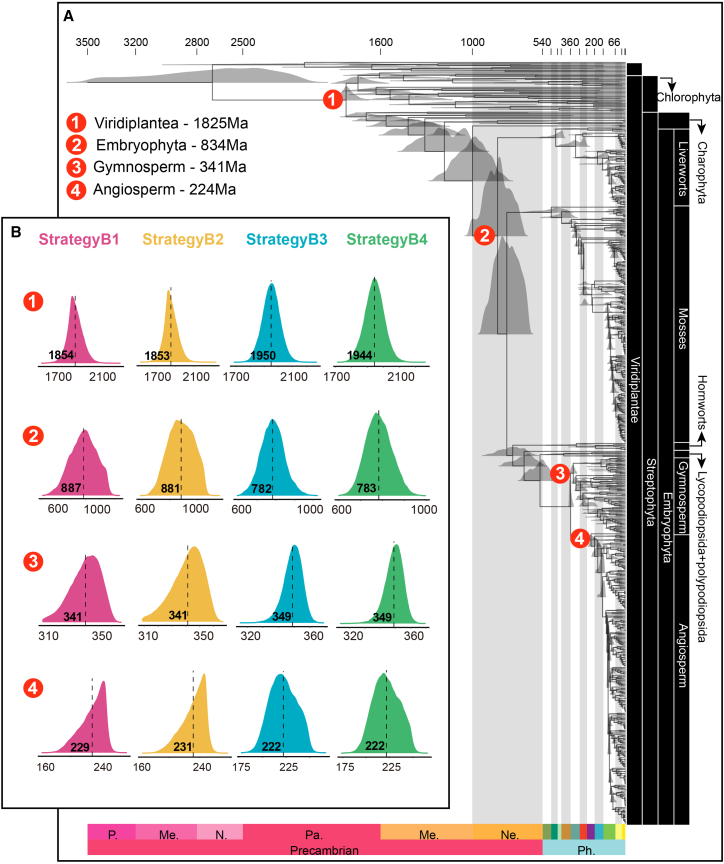
Time tree and posterior time distributions. **(A)** Detailed time tree for strategy A3 with posterior time distributions for each node. Red circles indicate four critical nodes, with mean posterior times displayed in the top left. Ph., Phanerozoic, (including Paleozoic, Mesozoic, and Cenozoic;); Ne., Neoproterozoic; Me., Mesoproterozoic; Pa., Paleoproterozoic. **(B)** Comparison of posterior time distributions for strategies B1–B4 at the four critical nodes. Dotted lines indicate mean posterior times.

Increasing fossil calibrations (B1 vs. A3) or removing three uncertain algal fossils (B2) produced negligible shifts in mean node ages (±<6 Ma). Partitioning codon positions (B3) and elevating σ^2^ heterogeneity (B4) reduced the mean age of Embryophyta by ∼100 Ma (to ∼780 Ma) but increased crown Viridiplantae estimates from ∼1 850 Ma to ∼1 950 Ma (Figure 4B; Supplemental Figures 14 and 15). This pattern may reflect high GC-content heterogeneity within chlorophytes, which display broad ecological and phenotypic diversity and are likely underestimated when heterogeneous models are not applied in molecular dating (Figure 4B).

For the sensitivity analysis, we tested the robustness of results to the number of calibrations by setting four gradients of fossil counts through sequential removal of calibration points (Supplemental Table 9). In strategy 1 (S1), we adopted 49 fossils, including all levels from outermost to innermost nodes. In strategy 2 (S2), we removed all fossils at or below the Order level, yielding 34. In strategy 3 (S3), we excluded all fossils at or below the Class level, leaving 23. In strategy 4 (S4), we excluded all fossils at or below the Phylum level, resulting in 13. Divergence-time estimation for these four strategies was conducted on the 207-species subtree used for testing alternative clock models (Supplemental Table 10). We then compared all inner node ages of S1 with those of S2, S3, and S4. In each case, points in the dot plots clustered closely along the diagonal (correlation >0.99), indicating consistent results across all four strategies. However, the absolute mean differences increased as the number of calibrations decreased, indicating that greater fossil reliability produces more robust dating outcomes (Supplemental Figure 17).

To provide an overview of the seven strategies, we summarized the inferred ages for key nodes across all approaches. Notably, strategy B3 produced a distinctly narrower credibility interval compared with strategies B1 and B2, indicating that partitioned data can greatly improve the precision of dating results (Table2). This conclusion was further supported by infinite-site plots, in which the regression coefficient (slope) reflects the degree of precision achieved with fossil calibrations. The slope for strategy B3 (0.7747) was clearly smaller than that of strategy B1 (1.2867) and strategy B2 (1.3297) (Supplemental Figure 16).

**Table 2:** Summary of inferred ages for key nodes across the different strategies.

Clade	95% Highest posterior density (Ma)						
	Strategy A (22 fossils)			Strategy B (53–55 fossils)			
	A1	A2	A3	B1	B2	B3	B4
Viridiplantae	1614–1944	1683–1969	1654–1915	1722–1997	1719–1998	1829–2071	1826–2062
Chlorophyta	1486–1874	1563–1897	1528–1851	1549–1893	1546–1894	1717–1954	1713–1944
Streptophyta	1372–1812	1508–1832	1469–1815	1510–1852	1508–1842	1617–1911	1666–1905
Embryophyta	488–518	690–1037	689–1029	715–1040	712–1038	664–908	676–899
Marchantiophyta	408–468	409–580	409–580	409–618	409–613	408–448	408–448
Marchantiopsida	229–391	229–400	229–411	229–402	230–416	228–354	228–334
Jungermanniopsida	102–204	105–204	104–201	169–289	169–299	167–195	167–196
Bryophyta	371–479	393–757	392–735	429–815	446–807	404–526	408–533
Takakiophytina	11–105	11–107	12–113	12–123	13–125	19–74	19–75
Andreaeophytina	7–45	7–45	7–44	5–52	8–49	8–28	8–28
Tetraphidopsida	22–291	22–293	24–296	29–292	25–207	48–282	47–283
Polytrichopsida	50–304	55–311	53–313	113–346	116–358	127–313	128–315
Bryopsida	285–351	287–372	287–381	288–378	291–409	290–323	290–324
Anthocerotaceae	94–384	102–538	101–531	232–625	234–641	276–458	280–461
Tracheophyta	424–451	513–866	521–852	525–867	516–856	571–802	583–797
Euphyllophyta	388–431	429–750	431–728	435–750	428–740	493–706	502–704
Spermatophyta	328–365	333–367	334–367	337–367	339–367	354–370	354–370
Acrogymnospermae	311–355	312–357	312–357	317–358	317–358	336–359	336–359
Angiospermae	180–249	181–249	182–249	192–252	196–252	197–247	197–247

## Discussion

### Comprehensive taxon sampling of mitochondrial data produces congruent phylogenies

Across the deep evolutionary history of green plants, spanning approximately 1 000 million years, mitochondrial genomes have preserved around 40 core protein-coding genes with lower mutation rates than those of nuclear genes (Wolfe et al., 1987; Drouin et al., 2008). This characteristic provides an advantage for resolving “deep-lineage” phylogenies. These highly conserved genes undergo uniparental inheritance, eliminating the effects of hybridization events that are common in nuclear genomes (Liu et al., 2014; Xue et al., 2022), thereby facilitating the retrieval of credible phylogenies for green plants using mitochondrial data. By specifically analyzing 39 core genes, we eliminated horizontally transferred genes, which are widespread in plant mitochondrial genomes. Previous studies have demonstrated that RNA editing, a common event in plant mitochondrial genomes, does not significantly affect phylogenetic reconstruction (Qiu et al., 2006, 2010). In this study, we analyzed 565 mitochondrial genomes representing all major green plant lineages to identify factors within mitochondrial genomes that could influence the accuracy of phylogenetic inference. The results from six partitioning schemes, ML analyses of three data types, and both homogeneous and heterogeneous Bayesian inference largely converged on a consistent phylogenetic backbone for the nine major green plant lineages, with the exception of chlorophytes, which were identified as paraphyletic under ExaBayes and P4 models (see above).

Our analyses revealed the presence of compositional heterogeneity and codon-usage bias in nt data, suggesting that positional heterogeneity may lead to a poor fit between molecular data and evolutionary models. However, application of six partition schemes yielded entirely congruent topologies, indicating that among-site heterogeneity exerted a minimal effect on phylogenetic inference. Liu et al. (2014) reported incongruent results for Bryophyta when using different datasets and models for mitochondrial data. Sousa et al. (2020) obtained different results by applying a non-stationary composition model to codon-degenerated and aa data, which partially aligned with nuclear-based phylogenies. Therefore, comprehensive sampling may sometimes eliminate the impact of systematic error.

Overall, our study provides new insights into comparing phylogenies inferred from mitochondrial versus nuclear genes and into exploring conflicting phylogenetic signals, particularly in the context of large-scale and dense sampling. Previous studies have highlighted many confounding factors in reconstructing green plant phylogeny, including variability sorting, codon degeneracy, nt data analyzed under non-stationary composition models, heterogeneous rate models, and coalescence of large gene trees. However, in practice, complex heterogeneous models are typically restricted to smaller datasets because of their high computational demands. For large-scale datasets with dense sampling, such as those from the 1KP project, only homogeneous and coalescent models have been used. In our study, we first confirmed the widespread presence of compositional heterogeneity in mitochondrial data, then adopted a heterogeneous model and implemented Bayesian methods to infer the phylogeny of the green lineage with extensive mitochondrial sampling.

Although our analyses produced largely congruent phylogenies with those inferred from nuclear genes, considerable incongruence remains on two long-standing questions compared with the 1KP project and other nuclear-based studies: (1) whether Zygnematophyceae or Charophyceae is the sister group of Embryophyta and (2) whether bryophytes are monophyletic or paraphyletic (Supplemental Figure 5) (Wickett et al., 2014; Lemieux et al., 2016; Gitzendanner et al., 2018; Puttick et al., 2018; Cheng et al., 2019; One Thousand Plant Transcriptomes Initiative, 2019; Del Cortona et al., 2020; Harris et al., 2020; Zhang et al., 2020; Su et al., 2021; Harris et al., 2022; Yang et al., 2022). Our alternative topology tests—the AU test, the KH test, and the bp-RELL and c-ELW indices—all significantly rejected the hypotheses of bryophyte monophyly and Zygnematophyceae as sister to Embryophyta (Supplemental Table 7). In nuclear genomes, frequent duplication events (tandem, chromosomal, and whole-genome duplications) and subsequent gene loss can disrupt orthology. Strictly speaking, absolute single-copy orthology at broad evolutionary scales, such as across all Embryophyta or all Angiospermae, might no longer exist. Consequently, most studies have now adopted the compromise of employing low-copy orthologous genes (Yang et al., 2020; Zuntini et al., 2024). Therefore, it might be unsurprising that different low-copy gene sets have produced incongruent topologies. In contrast, mitochondrial genomes readily provide single-copy genes with clear orthological relationships, although certain biases remain present and must be modeled. For example, GC-content heterogeneity and codon-usage bias, both evident in our data, were addressed in the heterogeneous Bayes model. However, a critical biological factor that may cause conflicting signals should not be ignored: the distinct inheritance modes of mitochondria and nuclei. Phylogenetic relationships inferred from mitochondrial data represent uniparental evolution (predominantly maternal, occasionally paternal), while nuclear data incorporate hybridization events that reflect contributions from both parents along with allelic variation. Furthermore, genomes from different cellular compartments (nuclear, chloroplast, and mitochondrial) display very different mutation rates. For instance, nuclear genes have been reported to evolve more rapidly than chloroplast and mitochondrial genes (Wolfe et al., 1987; Palmer and Herbon, 1988b; Drouin et al., 2008). Such variation in mutation rates could contribute to conflicting phylogenetic signals between mitochondrial and nuclear datasets. Finally, hornwort mitochondrial genomes contain comparatively fewer protein-coding genes (19–23) than other land plants, which may explain the consistently low support values observed at this node in our analyses.

High-quality data play fundamental roles in phylogenetic reconstruction and divergence-time estimation, particularly concerning large-scale phylogenies with extensive taxon sampling. In certain cases, conserved sites may be disproportionately influenced by rapidly evolving sites, where most lineages retain conserved positions; other lineages, such as chlorophytes in our dataset, contain a large proportion of rapidly evolving sites. Nevertheless, excessive deletion of such sites may lead to an insufficient number of informative sites. Therefore, it is crucial to select datasets of appropriate scale, maintaining a balance between the number of genes (sites) and species, with careful consideration. Additionally, data evaluation—including assessment of compositional heterogeneity and codon-usage bias—should be conducted in advance to ensure a comprehensive understanding of the dataset.

### Different dating strategies support the Neoproterozoic origin of land plants

Given the uncertainties of the fossil record, we incorporated 55 high-quality fossil calibrations, 52 of which adhered to best-practices guidelines (Parham et al., 2012). We considered all major factors that could influence divergence-time estimates in molecular dating, including taxon sampling, molecular data, tree topology, clock model, clock partitioning, rate prior, time prior, and fossil calibrations. Previous studies have demonstrated that the topology of bryophytes (paraphyletic or monophyletic) rarely influences molecular dating results (Morris et al., 2018). Therefore, we used the fixed tree from our phylogenetic analysis as the input tree. Here, our primary focus was on the effects of fossil calibrations, data partitioning, and rate priors. First, over-constraining the maximum calibration bound may result in younger age estimates (Clarke et al., 2011; Hedges et al., 2018; Su et al., 2021). To address this, we tested alternative strategies for the maximum bound. When the maximum bound was relaxed from 515 Ma (strategy A1) to 1042 Ma (strategy A2), the estimated crown age of Embryophyta shifted from the late Cambrian to the late Neoproterozoic. This change can be explained by the interaction between fossil and molecular data, which was assessed by comparing effective prior and posterior distributions. The effective prior distribution was generated by running the MCMC process without molecular data (i.e., using only the phylogenetic tree and fossil calibrations), while the posterior distribution incorporated all three sources of information: phylogeny, fossil calibration, and molecular sequences. Overlap between posterior and effective prior distributions indicates that molecular data were overridden by fossil constraints. Harris et al. (2022) also relaxed the maximum bound for Embryophyta but with denser fossil sampling than in earlier studies, leading to a substantially younger age estimate in the Ediacaran (540–597 Ma). To test whether additional calibrations would similarly lower estimates of the crown ages of Embryophyta or Viridiplantae, we compared results using 55 fossils (B1) versus 52 (B2) but observed no differences. Microfossils from palynological samples containing the oldest tetrahedral tetrads assigned to the Dapingian Stage of the Middle Ordovician (496 Ma) were used by Morris et al. (2018), whereas macrofossils from the Nanfeng Formation assigned to the Neoproterozoic (940.4 Ma) were employed by Harris et al. (2022). We tested a soft minimum bound using the macrofossils (940.4 Ma) for crown Viridiplantae (strategy A3) but found no change in estimated divergence times. Finally, analyses of our dataset characteristics revealed compositional heterogeneity across taxa and among codon positions (Figure 1), prompting further tests with a partitioned dataset. Heterogeneous evolutionary rates may lead to older age estimates (Beaulieu et al., 2015; Nie et al., 2020); thus, we applied a rate prior with greater variation among species. Both partitioned data (strategy B3) and higher-heterogeneity rate priors (strategy B4) produced crown Embryophyta age estimates that were ∼100 Ma younger.

Considering the limited number of loci in mitochondrial genomes, we generated infinite-sites plots by comparing posterior mean times against 95% credibility intervals with a regression line constrained through the origin to determine whether additional molecular data would improve the analysis. An exact linear relationship would indicate perfectly informative sequences. A strong correlation between credibility interval and posterior mean time was evident in our data, with all *R*-squared values near 0.6 (Supplemental Figure 16), suggesting that sufficient informative loci were present. Notably, when the maximum bounds were changed, our inferred origin time of land plants (early Neoproterozoic to Tonian) remained consistent with that reported by Su et al. (2021). In contrast, Harris et al. (2022) suggested an Ediacaran (late Neoproterozoic) origin of land plants based on more than 60 fossils, attributing the discrepancy to differences in phylogenetic assignment of certain fossils rather than to the choice of maximum age calibration. This substantial incongruence warrants further exploration. Additionally, our estimates for the crown age of Viridiplantae were robust across strategies dating back to the Paleoproterozoic–Mesoproterozoic, consistent with a recent study involving extensive Chlorophyta sampling (Yang et al., 2023).

In conclusion, the establishment of a reliable phylogeny and timescale for biological species requires adjustments for multiple sources of uncertainty. In this study on the origin and evolution of land plants, we incorporated key factors, including codon bias, among-site rate variation, GC content, fossil reliability, and appropriate calibration strategies.

## Methods

### Taxon sampling strategy

We collected 318 published mitochondrial genomes from the National Center for Biotechnology Information (NCBI) database, representing major green plant lineages. To ensure comprehensive coverage, we also utilized 16 angiosperm mitochondrial genomes from Xue et al. (2022) and 228 from Liu et al. (2019). In cases where mitochondrial genomes were unavailable for lineages occupying key phylogenetic positions, we sequenced mitochondrial genomes from four species and retrieved 14 Illumina sequencing datasets from the NCBI Sequence Read Archive (SRA), then conducted assembly and annotation (see below). To ensure high-quality data, we excluded species with fewer than 15 mitochondrial genes because these could generate excessively long branches; 32 such species were in Chlorophyta. The final dataset comprised mitochondrial genomes from 565 species, categorized as follows: eight Rhodophyta (outgroup), 30 Chlorophyta, 11 Charophyta, 60 liverworts, 181 mosses, six hornworts, one Lycopodiopsida, four Polypodiopsida, 58 Acrogymnospermae, and 206 Angiospermae. Details of all species used in the study are provided in Supplemental Table 1.

### Sequencing data processing

#### Sequencing and assembly of mitochondrial genomes

To enhance and balance taxon sampling of early-diverging angiosperms, we sequenced mitochondrial genomes of *Brasenia schreberi*, *Euryale ferox*, *Kadsura longipedunculata*, and *Illicium verum*. Fresh leaf tissues were collected from the Institute of Botany, Jiangsu Province, Chinese Academy of Sciences, Nanjing, China. High-quality genomic DNA was extracted using a modified cetyltrimethylammonium bromide protocol, and DNA quantity was assessed using a Qubit fluorometer (Invitrogen, Carlsbad, CA, USA). Next-generation sequencing libraries were prepared with the Illumina TruSeq Nano DNA Library Preparation Kit (Illumina, San Diego, CA, USA). The pooled libraries were enriched and subjected to paired-end sequencing on an Illumina MiSeq platform using the 600-cycle v3 sequencing kit (Illumina). Raw reads from the four newly sequenced species, as well as the 14 retrieved SRA datasets, were filtered using Fastp (v1.0.1) (Chen et al., 2018).

To assemble and annotate the 18 mitochondrial genomes, we used the HybPiper pipeline (v1.3) (https://github.com/mossmatters/HybPiper) and selected 19 high-quality mitochondrial genomes; their protein-coding genes served as reference bait sequences. These bait sequences are available in the figshare repository (https://figshare.com/account/projects/238673/articles/28466192?file=52550894). Subsequently, all cleaned reads were mapped to the reference sequences using BWA (v0.7.12) (www.github.com/lh3/bwa) to ensure that each gene contained mapped reads. SPAdes (v3.15.5) (www.github.com/ablab/spades) was then employed to assemble the mapped reads into contigs. Exonerate (v2.4.0) (www.github.com/nathanweeks/exonerate) was utilized to align the assembled contigs against the reference protein sequences. Finally, the Python script “retrieve_sequences.py” (https://github.com/mossmatters/HybPiper/tree/master/hybpiper) was employed to extract genes.

#### Data preprocessing

After retrieving 547 public and assembling 18 mitochondrial genomes, we detected annotation errors in the predicted protein-coding genes. We thus emphasized prechecking the data, as outlined below, to ensure accuracy in subsequent analyses. Protein-coding genes from each orthologous family were first reordered according to lineage to identify problematic sequences. Each reordered mitochondrial gene was then imported into MEGA5 (Tamura et al., 2011) for visualization, where sequences were meticulously checked for reversions and frameshift errors. Pseudogenes were excluded based on gene length and verification of conserved domains using multiple sequence alignments generated with the “Muscle” module in MEGA5. Furthermore, based on multiple alignment results, protein sequence regions lacking conserved motifs, which indicated annotation errors, were removed. After correction, both nt and aa sequences for each gene were generated for subsequent analyses.

We employed MAFFT (v7.2) (Rozewicki et al., 2019) to perform multiple sequence alignments for each mitochondrial gene. Subsequently, alignment gaps were trimmed using trimAL (v1.5.0) (Capella-Gutiérrez et al., 2009) with the “-automated1” setting. Corresponding nt alignments were obtained from each aa alignment by removing ambiguous positions using TranslatorX (v14.0) (Abascal et al., 2010). In total, 39 mitochondrial single-gene nt and aa alignments were concatenated with the custom Python script “concatenated.py.” All nt and corresponding aa alignment matrices have been deposited in the figshare repository (https://figshare.com/account/projects/238673/articles/28466231).

#### Evaluation of characteristics within the nucleotide dataset

We estimated compositional heterogeneity and codon-usage bias by calculating GC content and RSCU, respectively, for the concatenated nt alignment matrix. To evaluate base composition differences among major green plant lineages at different codon positions (first, second, third, and all positions combined), we calculated GC content at each position using the “GC” function in the R package “Seqinr (v4.2-36)” (https://cran.r-project.org/web/packages/seqinr/index.html). RSCU values were calculated with the “uco” function in Seqinr, and codon-usage bias was further analyzed by PCA using the pacman package (v0.5.1) (https://cran.r-project.org/web/packages/pacman/index.html).

### Phylogenetic analyses

Both nt and aa matrices were utilized for ML tree inference. ML searches were conducted using the parallel version of RAxML v8.2 (Stamatakis, 2014), with 1000 rapid bootstraps. For nt analyses, we applied the GTR (general time reversible) substitution model with gamma-distributed rate variation and a proportion of invariable sites (“GTR + Γ + I”). For aa analyses, we used the JTT empirical model (identified as the best-fit model by ModelFinder) with the same parameters (“JTT + Γ + I”). Because different genes may follow distinct evolutionary histories and codon positions evolve at different rates, we tested six partition schemes based on gene loci and codon positions: (1) no partition; (2) two partitions (first and second codon positions combined as one, third codon position as another); (3) three partitions (each codon position across all genes as one partition); (4) 39 partitions (each gene locus as one partition); (5) 78 partitions (first and second codon positions of each gene combined as one partition, third codon position as another); and (6) 117 partitions (each codon position within each gene locus as a separate partition) (Supplemental Table 6). All partitioned ML trees were inferred using RAxML v8.2 with the same parameters, enabling estimation and optimization of alpha-shape parameters, GTR rates, and empirical base frequencies for each partition. FigTree (v1.4.4) (http://tree.bio.ed.ac.uk/software/figtree/) was employed to visualize all trees and compare phylogenetic relationships across the results.

The concatenated nt dataset was analyzed via Bayesian inference using ExaBayes (v1.4) (Aberer et al., 2014) and P4 (Foster, 2004). In ExaBayes, a single global branch-length parameter was constrained for all partitions (i.e., linked) and other parameters were set as unlinked; the default GTR substitution model was applied. Priors for all parameters were left at their default values, and four chains across two runs were executed in parallel. Convergence was monitored using ASDSF as the key stopping criterion. An initial number of generations was specified, and runs were extended until the ASDSF value dropped below 5%. Convergence was achieved after 2 900 000 generations. A final consensus tree with posterior probability support values and branch-length information was obtained by summarizing tree samples, with the first 25% of trees discarded as burn-in. In P4—a Python library for Bayesian phylogenetic analysis designed for heterogeneous models—parameters were allowed to vary across tree partitions. We used the node-discrete composition heterogeneity (NDCH) model with the GTR+G substitution model. Configuration files for both ExaBayes and P4 are archived in a GitHub repository.

### Divergence-time estimation

Molecular dating was performed with the MCMCTree algorithm implemented in PAML v4.9. We employed the approximate likelihood calculation method (dos Reis and Yang, 2011), incorporated in MCMCTree, to improve computational efficiency. Tracer (v1.7.2) (https://github.com/beast-dev/tracer) was utilized to visualize MCMC results, ensuring that all parameter effective sample sizes exceeded 100. PPs of clade support were estimated by sampling trees from the posterior distribution after discarding the first 25% of samples as burn-in. Each MCMC analysis was run twice to assess convergence. Comprehensive taxon sampling encompassed 565 species, representing approximately 0.12% of the ∼500 000 total green plant species (Su et al., 2021). The fixed tree derived from our phylogenetic analysis was used as the input tree. Our analyses primarily focused on evaluating the effects of calibration strategies, data partitioning, and rate priors.

#### Clock models and rate priors

To compare the performance of the two relaxed-clock models provided in MCMCTree—the autocorrelated-rates model and the independent-rates model—we used 32 *b* values to estimate the marginal likelihood of each model with the “stepping-stone” algorithm. For computational efficiency, we carefully selected a reduced dataset of 207 species, ensuring representation at the Order level with at least one species per Order and 2–4 species for Orders with fossil calibrations. This approach allowed us to assume that the relative fit of the two models would also apply to the complete dataset (Barba-Montoya et al., 2018). We employed the independent-rates model, which provided a better fit, because it is more appropriate for distantly related taxa in large-scale datasets of green plants (Nie et al., 2020). The overall mutation rate was estimated using the BASEML program in the PAML package with the GTR+Γ model, based on pairwise distance between *Arabidopsis thaliana* and *Brachythecium rivulare*; their divergence time is approximately 469 Ma (Morris et al., 2018). Consequently, the prior per-site substitution rate was regarded as 0.03 per 100 Ma. The “rgene_gamma” parameter incorporates this rate to define the gamma prior for locus rates, considering shape (*a*) and scale (*b*) parameters, as well as mean (*m)* and standard deviation (*s). With* *m* = 0.03 (approximating the BASEML estimate) and *s* = 0.03, we calculated *a* = (*m*/*s*)^2^ and *b* = *m*/*s*^2^. The resulting shape parameter was *a* = (0.03/0.03)^2^ = 1, and the scale parameter was *b* = 0.03/0.03^2^ ≈ 33.3333. Accordingly, the control file specified “rgene_gamma = 1 33.” The “sigma2_gamma” parameter represents the variance of the logarithm of the rate (σ^2^), reflecting variability of rates among branches and the degree of clock violation at each locus. Following Morris et al. (2018), this was specified as a gamma distribution with shape 1 and scale 10. We also evaluated the effects of among-branch rate variation in strategy B4 (see below).

Time priors were established through a birth–death process based on fossil calibrations. The “BDparas” parameter defines tree shape using three variables: birth rate (λ), death rate (μ), and sampling fraction (ρ). We specified “λ = 1, μ = 1, ρ = 0.0012” to generate a uniform kernel for branching times. We then conducted an MCMC analysis exclusively utilizing the specified topology and fossils, with “usedata = 0” indicating exclusion of sequence data. This setup produced a joint distribution—also referred to as the effective time prior—that encompassed all node ages. The approximate likelihood method does not directly incorporate molecular data into divergence-time estimation but instead relies on the gradient (*g*) and Hessian (*H*), which represent the first and second derivatives of the log-likelihood function, evaluated at the ML estimates of branch lengths. Accordingly, BASEML was employed to compute gradient and Hessian values for branch lengths.

#### Calibration strategies

In strategy A, we employed 22 fossil calibrations (Table 1 and Supplemental Table 8), each defined by a hard minimum bound and a soft maximum bound following a uniform distribution. Three approaches were designed to test the accuracy of dating results. In strategy A1, we applied a maximum age of 515 Ma to early Embryophyta nodes (consistent with Morris et al., 2018) and assigned uniform priors to all calibration nodes, with a hard minimum bound (*p*L = 1e−300) and a soft maximum bound (*p*U = 0.025). In strategy A2, we relaxed the maximum age for early Embryophyta nodes to 1042 Ma (consistent with Su et al., 2021), while maintaining all other prior distributions from strategy A1. In strategy A3, we adjusted the minimum fossil age of crown Viridiplantae from 469 Ma (microfossils of the oldest tetrahedral tetrads, Dapingian Stage) to 940 Ma (macrofossils from the Nanfen Formation). Additionally, considering the controversy and uncertainty surrounding this node, we applied a uniform prior with a soft minimum bound (*p*L = 0.1) and a soft maximum bound (*p*U = 0.025) for crown Viridiplantae, while retaining the same settings as strategy A1 for all other nodes. The root age was set at <3500 Ma, referencing the earliest known fossil evidence.

In strategy B (Supplemental Table 8), four additional approaches were applied to evaluate the effects of fossil quantity, data partitioning, and variation in among-branch rates. Strategy B1 incorporated 33 additional fossil calibrations for critical nodes, increasing the total to 55 fossils. In strategy B2, three uncertain algal fossils were excluded—Prasinophyceae (Pterospermella, 1200 Ma), *Chlorophyceae* (*Palaeastrum*, 750 Ma), and Charophyceae (*Trochiliscus* sp., 405 Ma)—resulting in 52 fossils. Strategy B3 (partitioned data) divided the concatenated nt dataset into three partitions, with each codon position regarded as a separate partition. In strategy B4 (a rate prior with greater among-lineage heterogeneity), the rate-drift parameter (σ^2^) was adjusted to follow a distribution of σ^2^ ∼ (10,10).

## Data and code availability

Raw sequencing reads generated for the assembly of mitochondrial genomes from four basal angiosperm species have been deposited in NCBI under accession PRJNA1228570. The bait sequence file used for extracting protein-coding genes from assemblies, along with the final concatenated alignments, has been deposited in figshare (https://figshare.com/account/home#/projects/238673). All custom Python scripts for data processing, as well as configuration files for ExaBayes, P4, and MCMCTree, are archived in our GitHub repository (https://github.com/whosya/Mito_phylogeny).

## Funding

J.-Y.X. was supported by the 10.13039/501100001809National Natural Science Foundation of China (32570265), the 10.13039/501100012226Fundamental Research Funds for the Central Universities (RENCAI2025034 and KYCXJC2025002), the 10.13039/501100012274State Key Laboratory of Palaeobiology and Stratigraphy, Nanjing Institute of Geology and Palaeontology, 10.13039/501100002367CAS (213124), and the National Administration of Traditional Chinese Medicine High-Level Key Discipline Construction Project (zyyzdxk-2023293). Z.L. was supported by the Junior Research Project of 10.13039/501100003130FWO (G0ADO25N) and the Special Research Grant from 10.13039/501100004385Ghent University (BOF.BAF.2024.0889.01). Y.V.d.P. was supported by the 10.13039/501100000781European Research Council (ERC) under the European Union’s Horizon 2020 Research and Innovation Program (833522) and by 10.13039/501100004385Ghent University (Methusalem funding, BOF.MET.2021.0005.01).

## Acknowledgments

Special thanks are extended to Hengchi Chen for his valuable suggestions on divergence-time analyses. No conflict of interest declared.

## Author contributions

J.-Y.X., G.S., Y.V.d.P., and Z.L. conceived the study and revised the manuscript; C.-A.Y., J.-Y.X., G.S., and S.-Y.H. collected the data; G.S. verified fossil calibrations; S.-Y.H. conducted the analyses and wrote the manuscript.

## Supplemental information

Supplemental Data 1. Supplemental Tables 1–10

Document S2. Article plus supplemental information

## References

[bib1] Abascal F., Zardoya R., Telford M.J. (2010). TranslatorX: multiple alignment of nucleotide sequences guided by amino acid translations. Nucleic Acids Res..

[bib2] Aberer A.J., Kobert K., Stamatakis A. (2014). ExaBayes: Massively Parallel Bayesian Tree Inference for the Whole-Genome Era. Mol. Biol. Evol..

[bib3] Beaulieu J.M., O’Meara B.C., Crane P., Donoghue M.J. (2015). Heterogeneous Rates of Molecular Evolution and Diversification Could Explain the Triassic Age Estimate for Angiosperms. Syst. Biol..

[bib4] Barba-Montoya J., Dos Reis M., Schneider H., Donoghue P.C.J., Yang Z. (2018). Constraining uncertainty in the timescale of angiosperm evolution and the veracity of a Cretaceous Terrestrial Revolution. New Phytol..

[bib5] Becker B., Marin B. (2009). Streptophyte algae and the origin of embryophytes. Ann. Bot..

[bib6] Beckert S., Steinhauser S., Muhle H., Knoop V. (1999). A molecular phylogeny of bryophytes based on nucleotide sequences of the mitochondrialnad5 gene. Pl. Syst. Evol..

[bib7] Blank C.E. (2013). Origin and early evolution of photosynthetic eukaryotes in freshwater environments: reinterpreting proterozoic paleobiology and biogeochemical processes in light of trait evolution. J. Phycol..

[bib8] Bowe L.M., Coat G., dePamphilis C.W. (2000). Phylogeny of seed plants based on all three genomic compartments: Extant gymnosperms are monophyletic and Gnetales’ closest relatives are conifers. Proc. Natl. Acad. Sci. USA.

[bib9] Capella-Gutiérrez S., Silla-Martínez J.M., Gabaldón T. (2009). trimAl: a tool for automated alignment trimming in large-scale phylogenetic analyses. Bioinformatics.

[bib10] Chen S., Zhou Y., Chen Y., Gu J. (2018). fastp: an ultra-fast all-in-one FASTQ preprocessor. Bioinformatics.

[bib11] Cheng S., Xian W., Fu Y., Marin B., Keller J., Wu T., Sun W., Li X., Xu Y., Zhang Y. (2019). Genomes of Subaerial Zygnematophyceae Provide Insights into Land Plant Evolution. Cell.

[bib12] Civáň P., Foster P., Embley T., Séneca A., Cox C. (2014). Analyses of Charophyte Chloroplast Genomes Help Characterize the Ancestral Chloroplast Genome of Land Plants. Genome Biol. Evol..

[bib13] Clarke J.T., Warnock R.C.M., Donoghue P.C.J. (2011). Establishing a time-scale for plant evolution. New Phytol..

[bib14] Cox C.J. (2018). Land Plant Molecular Phylogenetics: A Review with Comments on Evaluating Incongruence Among Phylogenies. Crit. Rev. Plant Sci..

[bib15] Cox C.J., Li B., Foster P.G., Embley T.M., Civán P. (2014). Conflicting Phylogenies for Early Land Plants are Caused by Composition Biases among Synonymous Substitutions. Syst. Biol..

[bib16] de Vries J., Archibald J.M. (2018). Plant evolution: landmarks on the path to terrestrial life. New Phytol..

[bib17] Del Cortona A., Jackson C.J., Bucchini F., Van Bel M., D’hondt S., Škaloud P., Delwiche C.F., Knoll A.H., Raven J.A., Verbruggen H. (2020). Neoproterozoic origin and multiple transitions to macroscopic growth in green seaweeds. Proc. Natl. Acad. Sci. USA.

[bib18] Del Vasto M., Figueroa-Martinez F., Featherston J., González M.A., Reyes-Prieto A., Durand P.M., Smith D.R. (2015). Massive and Widespread Organelle Genomic Expansion in the Green Algal Genus Dunaliella. Genome Biol. Evol..

[bib19] Donoghue P.C.J., Harrison C.J., Paps J., Schneider H. (2021). The evolutionary emergence of land plants. Curr. Biol..

[bib20] Drouin G., Daoud H., Xia J. (2008). Relative rates of synonymous substitutions in the mitochondrial, chloroplast and nuclear genomes of seed plants. Mol. Phylogenet. Evol..

[bib21] Forrest L.L., Davis E.C., Long D.G., Crandall-Stotler B.J., Clark A., Hollingsworth M.L. (2006). Unraveling the evolutionary history of the liverworts (Marchantiophyta): multiple taxa, genomes and analyses. Bryologist.

[bib22] Foster P.G. (2004). Modeling Compositional Heterogeneity. Syst. Biol..

[bib23] Fučíková K., Lewis P.O., Lewis L.A. (2014). Widespread desert affiliation of trebouxiophycean algae (Trebouxiophyceae, Chlorophyta) including discovery of three new desert genera. Phycol. Res..

[bib25] Gray D.W., Lewis L.A., Cardon Z.G. (2007). Photosynthetic recovery following desiccation of desert green algae (Chlorophyta) and their aquatic relatives. Plant Cell Environ..

[bib26] Gitzendanner M.A., Soltis P.S., Wong G.K.-S., Ruhfel B.R., Soltis D.E. (2018). Plastid phylogenomic analysis of green plants: A billion years of evolutionary history. Am. J. Bot..

[bib27] Harris B.J., Harrison C.J., Hetherington A.M., Williams T.A. (2020). Phylogenomic Evidence for the Monophyly of Bryophytes and the Reductive Evolution of Stomata. Curr. Biol..

[bib28] Harris B.J., Clark J.W., Schrempf D., Szöllősi G.J., Donoghue P.C.J., Hetherington A.M., Williams T.A. (2022). Divergent evolutionary trajectories of bryophytes and tracheophytes from a complex common ancestor of land plants. Nat. Ecol. Evol..

[bib29] Hedges S.B., Tao Q., Walker M., Kumar S. (2018). Accurate timetrees require accurate calibrations. Proc. Natl. Acad. Sci. USA.

[bib30] Hu H., Sun P., Yang Y., Ma J., Liu J. (2023). Genome-scale angiosperm phylogenies based on nuclear, plastome, and mitochondrial datasets. J. Integr. Plant Biol..

[bib31] Holzinger A., Karsten U. (2013). Desiccation stress and tolerance in green algae: consequences for ultrastructure, physiological and molecular mechanisms. Front. Plant Sci..

[bib32] Knauth L.P., Kennedy M.J. (2009). The late Precambrian greening of the Earth. Nature.

[bib33] Knoop V. (2004). The mitochondrial DNA of land plants: peculiarities in phylogenetic perspective. Curr. Genet..

[bib34] Leliaert F., Tronholm A., Lemieux C., Turmel M., DePriest M.S., Bhattacharya D., Karol K.G., Fredericq S., Zechman F.W., Lopez-Bautista J.M. (2016). Chloroplast phylogenomic analyses reveal the deepest-branching lineage of the Chlorophyta, Palmophyllophyceae class. nov. Sci. Rep..

[bib35] Lemieux C., Otis C., Turmel M. (2016). Comparative Chloroplast Genome Analyses of Streptophyte Green Algae Uncover Major Structural Alterations in the Klebsormidiophyceae, Coleochaetophyceae and Zygnematophyceae. Front. Plant Sci..

[bib36] Lewis L.A., Flechtner V.R. (2002). Green algae (Chlorophyta) of desert microbiotic crusts: diversity of North American taxa. Taxon.

[bib37] Lewis L.A., McCourt R.M. (2004). Green algae and the origin of land plants. Am. J. Bot..

[bib38] Li X., Hou Z., Xu C., Shi X., Yang L., Lewis L.A., Zhong B. (2021). Large Phylogenomic Data sets Reveal Deep Relationships and Trait Evolution in Chlorophyte Green Algae. Genome Biol. Evol..

[bib39] Li H.-T., Yi T.-S., Gao L.-M., Ma P.-F., Zhang T., Yang J.-B., Gitzendanner M.A., Fritsch P.W., Cai J., Luo Y. (2019). Origin of angiosperms and the puzzle of the Jurassic gap. Nat. Plants.

[bib40] Liu Y., Cox C.J., Wang W., Goffinet B. (2014). Mitochondrial Phylogenomics of Early Land Plants: Mitigating the Effects of Saturation, Compositional Heterogeneity, and Codon-Usage Bias. Syst. Biol..

[bib41] Liu Y., Johnson M.G., Cox C.J., Medina R., Devos N., Vanderpoorten A., Hedenäs L., Bell N.E., Shevock J.R., Aguero B. (2019). Resolution of the ordinal phylogeny of mosses using targeted exons from organellar and nuclear genomes. Nat. Commun..

[bib42] Lutzoni F., Nowak M.D., Alfaro M.E., Reeb V., Miadlikowska J., Krug M., Arnold A.E., Lewis L.A., Swofford D.L., Hibbett D. (2018). Contemporaneous radiations of fungi and plants linked to symbiosis. Nat. Commun..

[bib43] McCourt R.M., Lewis L.A., Strother P.K., Delwiche C.F., Wickett N.J., de Vries J., Bowman J.L. (2023). Green land: Multiple perspectives on green algal evolution and the earliest land plants. Am. J. Bot..

[bib44] Mennes C.B., Smets E.F., Moses S.N., Merckx V.S.F.T. (2013). New insights in the long-debated evolutionary history of Triuridaceae (Pandanales). Mol. Phylogenet. Evol..

[bib45] Mikhailyuk T., Lukešová A., Glaser K., Holzinger A., Obwegeser S., Nyporko S., Friedl T., Karsten U. (2018). New Taxa of Streptophyte Algae (Streptophyta) from Terrestrial Habitats Revealed Using an Integrative Approach. Protist.

[bib46] Morris J.L., Puttick M.N., Clark J.W., Edwards D., Kenrick P., Pressel S., Wellman C.H., Yang Z., Schneider H., Donoghue P.C.J. (2018). The timescale of early land plant evolution. Proc. Natl. Acad. Sci. USA.

[bib47] Nedelcu A.M., Lee R.W., Lemieux C., Gray M.W., Burger G. (2000). The Complete Mitochondrial DNA Sequence of Scenedesmus obliquus Reflects an Intermediate Stage in the Evolution of the Green Algal Mitochondrial Genome. Genome Res..

[bib48] Nie Y., Foster C.S.P., Zhu T., Yao R., Duchêne D.A., Ho S.Y.W., Zhong B. (2020). Accounting for Uncertainty in the Evolutionary Timescale of Green Plants Through Clock-Partitioning and Fossil Calibration Strategies. Syst. Biol..

[bib49] One Thousand Plant Transcriptomes Initiative (2019). One thousand plant transcriptomes and the phylogenomics of green plants. Nature.

[bib50] Palmer J.D., Herbon L.A. (1988). Plant mitochondrial DNA evolved rapidly in structure, but slowly in sequence. J. Mol. Evol..

[bib51] Palmer J.D., Herbon L.A. (1988). Plant mitochondrial DNA evolved rapidly in structure, but slowly in sequence. J. Mol. Evol..

[bib52] Parham J.F., Donoghue P.C.J., Bell C.J., Calway T.D., Head J.J., Holroyd P.A., Inoue J.G., Irmis R.B., Joyce W.G., Ksepka D.T. (2012). Best Practices for Justifying Fossil Calibrations. Syst. Biol..

[bib53] Puttick M.N., Morris J.L., Williams T.A., Cox C.J., Edwards D., Kenrick P., Pressel S., Wellman C.H., Schneider H., Pisani D., Donoghue P.C.J. (2018). The Interrelationships of Land Plants and the Nature of the Ancestral Embryophyte. Curr. Biol..

[bib54] Qiu Y.-L. (2008). Phylogeny and evolution of charophytic algae and land plants. J. Systemat. Evol..

[bib55] Qiu Y.-L., Mishler B.D. (2024). Relationships Among the Bryophytes and Vascular Plants: A Case Study in Deep-Time Reconstruction. Diversity.

[bib56] Qiu Y.-L., Palmer J.D., Qiu Y.-L., Palmer J.D. (1999). Phylogeny of early land plants: insights from genes and genomes. Trends Plant Sci..

[bib57] Qiu Y.-L., Lee J., Bernasconi-Quadroni F., Soltis D.E., Soltis P.S., Zanis M., Zimmer E.A., Chen Z., Savolainen V., Chase M.W. (1999). The earliest angiosperms: evidence from mitochondrial, plastid and nuclear genomes. Nature.

[bib58] Qiu Y.-L., Li L., Wang B., Chen Z., Knoop V., Groth-Malonek M., Dombrovska O., Lee J., Kent L., Rest J. (2006). The deepest divergences in land plants inferred from phylogenomic evidence. Proc. Natl. Acad. Sci. USA.

[bib59] Qiu Y.-L., Li L., Wang B., Xue J.-Y., Hendry T.A., Li R.-Q., Brown J.W., Liu Y., Hudson G.T., Chen Z.-D. (2010). Angiosperm phylogeny inferred from sequences of four mitochondrial genes. J. Systemat. Evol..

[bib60] Qiu Y.-L., Taylor A.B., McMANUS H.A. (2012). Evolution of the life cycle in land plants: QIU et al.: Plant life cycle evolution. J. Syst. Evol..

[bib61] dos Reis M., Yang Z. (2011). Approximate Likelihood Calculation on a Phylogeny for Bayesian Estimation of Divergence Times. Mol. Biol. Evol..

[bib62] Rozewicki J., Li S., Amada K.M., Standley D.M., Katoh K. (2019). MAFFT-DASH: integrated protein sequence and structural alignment. Nucleic Acids Res..

[bib63] Ruhfel B.R., Gitzendanner M.A., Soltis P.S., Soltis D.E., Burleigh J.G. (2014). From algae to angiosperms–inferring the phylogeny of green plants (Viridiplantae) from 360 plastid genomes. BMC Evol. Biol..

[bib64] Sousa F., Foster P.G., Donoghue P.C.J., Schneider H., Cox C.J. (2019). Nuclear protein phylogenies support the monophyly of the three bryophyte groups (Bryophyta Schimp.). New Phytol..

[bib65] Sousa F., Civáň P., Brazão J., Foster P.G., Cox C.J. (2020). The mitochondrial phylogeny of land plants shows support for Setaphyta under composition-heterogeneous substitution models. PeerJ.

[bib67] Stull G.W., Qu X.-J., Parins-Fukuchi C., Yang Y.-Y., Yang J.-B., Yang Z.-Y., Hu Y., Ma H., Soltis P.S., Soltis D.E. (2021). Gene duplications and phylogenomic conflict underlie major pulses of phenotypic evolution in gymnosperms. Nat. Plants.

[bib68] Stamatakis A. (2014). RAxML version 8: a tool for phylogenetic analysis and post-analysis of large phylogenies. Bioinformatics.

[bib69] Strother P.K., Battison L., Brasier M.D., Wellman C.H. (2011). Earth’s earliest non-marine eukaryotes. Nature.

[bib70] Su D., Yang L., Shi X., Ma X., Zhou X., Hedges S.B., Zhong B. (2021). Large-scale phylogenomic analyses reveal the monophyly of bryophytes and Neoproterozoic origin of land plants. Mol. Biol. Evol..

[bib71] Tamura K., Peterson D., Peterson N., Stecher G., Nei M., Kumar S. (2011). MEGA5: Molecular Evolutionary Genetics Analysis Using Maximum Likelihood, Evolutionary Distance, and Maximum Parsimony Methods. Mol. Biol. Evol..

[bib72] Turmel M., Otis C., Lemieux C. (2013). Tracing the Evolution of Streptophyte Algae and Their Mitochondrial Genome. Genome Biol. Evol..

[bib73] Wickett N., Mirarab S., Nguyen N.-P., Warnow T., Carpenter E., Matasci N., Ayyampalayam S., Barker M.S., Burleigh J., Gitzendanner M. (2014). Phylotranscriptomic analysis of the origin and early diversification of land plants. Proc. Natl. Acad. Sci. USA.

[bib74] Königlich-böhmische Gesellschaft der Wissenschaften (1874). Sitzungsberichte der königl. böhmischen Gesellschaft der Wissenschaften in Prag.p. 0 Königlich-böhmische Gesellschaft der Wissenschaften.

[bib75] Wolfe K.H., Li W.H., Sharp P.M. (1987). Rates of nucleotide substitution vary greatly among plant mitochondrial, chloroplast, and nuclear DNAs. Proc. Natl. Acad. Sci. USA.

[bib76] Yang E.C., Boo S.M., Bhattacharya D., Saunders G.W., Knoll A.H., Fredericq S., Graf L., Yoon H.S. (2016). Divergence time estimates and the evolution of major lineages in the florideophyte red algae. Sci. Rep..

[bib77] Xue J.Y., Dong S.S., Wang M.Q., Song T.Q., Zhou G.C., Li Z., Van de Peer Y., Shao Z.Q., Wang W., Chen M. (2022). Mitochondrial genes from 18 angiosperms fill sampling gaps for phylogenomic inferences of the early diversification of flowering plants. J. Syst. Evol..

[bib78] Yang T., Sahu S.K., Yang L., Liu Y., Mu W., Liu X., Strube M.L., Liu H., Zhong B. (2022). Comparative analyses of 3,654 plastid genomes unravel insights into evolutionary dynamics and phylogenetic discordance of green plants. Front. Plant Sci..

[bib79] Yang Y., Sun P., Lv L., Wang D., Ru D., Li Y., Ma T., Zhang L., Shen X., Meng F. (2020). Prickly waterlily and rigid hornwort genomes shed light on early angiosperm evolution. Nat. Plants.

[bib80] Yang Z., Ma X., Wang Q., Tian X., Sun J., Zhang Z., Xiao S., De Clerck O., Leliaert F., Zhong B. (2023). Phylotranscriptomics unveil a Paleoproterozoic-Mesoproterozoic origin and deep relationships of the Viridiplantae. Nat. Commun..

[bib81] Zhang N., Wen J., Zimmer E.A. (2016). Another look at the phylogenetic position of the grape order Vitales: Chloroplast phylogenomics with an expanded sampling of key lineages. Mol. Phylogenet. Evol..

[bib82] Zhang L., Huang C.-H., Zhang G., Zhang C., Zhao Y., Huang J., Guo J., Cheng L., Zhang T., Ma H. (2025). Nuclear Phylogenomics of Angiosperms and Evolutionary Implications. Diversity.

[bib83] Zhang J., Fu X.X., Li R.Q., Zhao X., Liu Y., Li M.H., Zwaenepoel A., Ma H., Goffinet B., Guan Y.L. (2020). The hornwort genome and early land plant evolution. Nat. Plants.

[bib84] Zhong B., Xi Z., Goremykin V.V., Fong R., Mclenachan P.A., Novis P.M., Davis C.C., Penny D. (2014). Streptophyte Algae and the Origin of Land Plants Revisited Using Heterogeneous Models with Three New Algal Chloroplast Genomes. Mol. Biol. Evol..

[bib85] Zuntini A.R., Carruthers T., Maurin O., Bailey P.C., Leempoel K., Brewer G.E., Epitawalage N., Françoso E., Gallego-Paramo B., McGinnie C. (2024). Phylogenomics and the rise of the angiosperms. Nature.

