# Supplementary material for: Comprehensive sampling of mitochondrial genomes substantiates the Neoproterozoic origin of land plants
Source: Plant Commun. 2025 Sep 5;6(11):101497. doi: 10.1016/j.xplc.2025.101497 (PMC12785157; doi:10.1016/j.xplc.2025.101497)
Supplement: Document S1. Supplemental Figures 1–17 [file mmc1.pdf]

Plant Communications, Volume *6*

Supplemental information

Comprehensive sampling of mitochondrial genomes substantiates the Neoproterozoic origin of land plants

Shuai-Ya Hu, Gongle Shi, Cheng-Ao Yang, Yves Van de Peer, Zhen Li, and Jia-Yu Xue

Comprehensive sampling from mitochondrial genomes substantiates the Neoproterozoic origin of land plants

Shuai-Ya Hu[1,2,3], Gongle Shi[4], Cheng-Ao Yang[1], Yves Van de Peer[1,2,3,5,*], Zhen Li[2,3,*], Jia-Yu Xue[1,*]

[1]College of Horticulture, Bioinformatics Center, Academy for Advanced Interdisciplinary Studies, Nanjing Agricultural University, Nanjing 210095, China

[2]Department of Plant Biotechnology and Bioinformatics, Ghent University, B-9052 Ghent, Belgium

[3]VIB-UGent Center for Plant Systems Biology, B-9052 Ghent, Belgium

[4]State Key Laboratory of Palaeobiology and Stratigraphy, Nanjing Institute of Geology and Palaeontology, Chinese Academy of Sciences, Nanjing, China

[5]Department of Biochemistry, Genetics and Microbiology, University of Pretoria, Pretoria 0028, South Africa

*To whom correspondence should be addressed: xuejy@njau.edu.cn (J.Y.X.), zhen.li@psb.vib-ugent.be (Z.L.), or yves.vandepeer@psb.vib-ugent.be (Y.V.d.P.)

Supplemental materials

Here we supplement the justifications on two key, controversial calibration points, and the reasons for different setting of their maximum constraint bound or minimum constraint bound in different strategies. We will not describe the details of different dating strategies Because they have already been elaborated in detail in the main text. There are four controversial fossil calibration need to be clarified:

First, the different maximum bound for early Embryophyta node ages constraint used in strategy A1 and strategy A2. Morris et al. (2018) adopted an age of 515.5MA based on Cryptospores that have recently been reported from the Rome Formation that underlies the Conasauga Group and is correlated to the *Olenellus* trilobite Biozone of Stage 4 of Series 2, the latest part of the Early Cambrian. The base of the Olenellus Biozone is dated to approximately 515.5 Ma. This choice is based on the criteria that a soft maximum age constraint for the Marchantiophyta crown group must encompass all Cryptospore records. Cryptospores from the Rome Formation is so far the oldest record as long as no new Cryptospores report, therefore, 515.5MA adopted here is relative conservative. So, we also resort to another much older maximum bound - 1042MA justified by Clarke et al. (2011) based on the following criteria: an arbitrary but objective and precise date can be obtained from Precambrian sediments of the Torridon Group, Scotland, which represent an environment in which embryophytes would be expected to have flourished were their lineage established. The sequences have been investigated palynologically but yield no evidence of spores or other remains with possible embryophyte affinities (Strother et al., 2011). A direct date of 994Ma±48Myr was obtained from the Diabaig Formation of the Torridon group (Turnbull et al., 1996), and thus 1042MA can provide a soft maximum constraint for crown Embryophyta. A detailed justification about why choosing 1042MA as soft maximum constraint for crown Embryophyta was described by Clarke et al. (2011). Since the two types of justification both make sense, we accommodate them into two (sub)strategies, managing to explore how they respectively interact with molecular data.

Second, two different fossils used as minimum bound for crown Viridiplantae constraint by Morris et al. (2018) and Harris et al. (2022), respectively. Morris et al. (2018) adopted a minimum age constraint of 469 MA which is based on *Tetrahedraletes* cf. *medinensis*, a palynological sample containing the oldest

tetrahedral tetrad, collected from strata in the transition between the upper part of the Zanjón Formation and the Laja Morada Member of the Labrado Formation. This sample was assigned to the Dapinigian Stage of the Middle Ordovician, based on the presence of the chitinozoan Lagenochitina combazi, a biostratigraphical marker of the L. combazi Biozone of southern China (Chen et al., 2009). The top of the combazi Biozone has been correlated closely to the boundary between the chitinozoan D. ornensis and B. henryi Biozones of Northern Gondwana (Chen et al., 2009; Paris et al., 1990). Dated at 469 MA (Cooper et al., 2012), this boundary is the youngest possible age for the top of the correlated L. combazi Biozone, and thus the minimum age of the tetrahedral tetrad record from Argentina, 1 Myr after the beginning of the Dapingian Stage/ Middle Ordovician. However, very recent studies claimed a much older age for minimum bound constraint of crown Viridiplantae node based on a new justified fossil *Proterocladus antiquus* (Tang et al., 2020; Harris et al., 2022). *Proterocladus antiquus* is a kind of Macrofossils from the Nanfen Formation, Northern China. A sill in the overlying Qiaotou Formation, which overlies the Nanfeng Formation, has a zircon secondary-ion mass spectrometry U-Pb age of 947.8 MA ± 7.4 Myr (Zhao et al., 2019). Thus, the minimum age of the Nanfeng Formation can be established on the minimum age interpretation of this sill viz. 940.4 MA, although it is presumably significantly older. Theoretically speaking, one should always adopt the newest fossils as the best calibration, but we still want to explore if a smaller setting of minimum bound would largely influence the estimated time scale (i.e., the sencitivity). So, 469 Ma was used in strategy A2 and 940.4 MA was used in strategy A3 for comparison.

References

Clarke, J.T., Warnock, R.C.M., and Donoghue, P.C.J. (2011). Establishing a time-scale for plant evolution. *New Phytologist* **192**: 266-301.

Chen, X., Paris, F., Wang, X., Zhang, M. (2009). Early and Middle Ordovician chitinozoans from the Dapingian type sections, Yichang area, China. *Review of Palaeobotany and Palynology* **153**: 310–330.

Cooper, R.A., Sadler, P.M. (2012). The Ordovician Period. In: Gradstein, F.M., Ogg, J.G., Schmitz, M.D., Ogg, G.M. (eds.). *The Geological Time Scale 2012* **2**: 489–523.

Harris, B., Clark, J., Schrempf, D., Szollosi, G., Donoghue, P., Hetherington, A., and Williams, T. (2022b). Divergent evolutionary trajectories of bryophytes and

tracheophytes from a complex common ancestor of land plants. *Nature Ecology & Evolution* **6**:1–10.

Morris, J. L., Puttick, M. N., Clark, J. W., Edwards, D., Kenrick, P., Pressel, S., Wellman, C. H., Yang, Z., Schneider, H., and Donoghue, P. C. J. (2018). The timescale of early land plant evolution. *Proceedings of the National Academy of Sciences* **115**: E2274–E2283.

Peng, S., Babcock, L.E., Cooper, R.A. (2012). The Cambrian Period. In: Gradstein, F.M., Ogg, J.G., Schmitz, M.D., Ogg, G.M. (eds.), *The Geological Time Scale 2012* **2**: 437–488.

Paris, F. 1990. The Ordovician chitinozoan biozones of the Northern Gondwana Domain. *Review of Palaeobotany and Palynology* **66**:190–191.

Strother, P.K., Battison, L., Brasier, M.D., Wellman, C.H. (2011). Earth's earliest non-marine eukaryotes. *Nature* **473**: 505–509.

Strother, P.K. (2016). Systematics and evolutionary significance of some new cryptospores from the Cambrian of eastern Tennessee, USA. *Review of Palaeobotany and Palynology* **227**: 28–41.

Turnbull, M.J.M., Whitehouse, M.J., Moorbath, S. (1996). New isotopic age determinations for the Torridonian, NW Scotland. *Journal of the Geological Society* **153**: 955–964.

Tang Q., Pang K., Yuan X., Xiao S. (2020). A one-billion-year-old multicellular chlorophyte. *Nature Ecology & Evolution* **4**:543-549.

Zhao H., Zhang S., Ding J., Chang L., Ren Q., Li H., Yang T., Wu H. (2019). New geochronologic and paleomagnetic results from early Neoproterozoic mafic sills and late Mesoproterozoic to early Neoproterozoic successions in the eastern North China Craton, and implications for the reconstruction of Rodinia. *GSA Bulletin* **132**: 739-766.

Supplemental Figures

Main Workflow

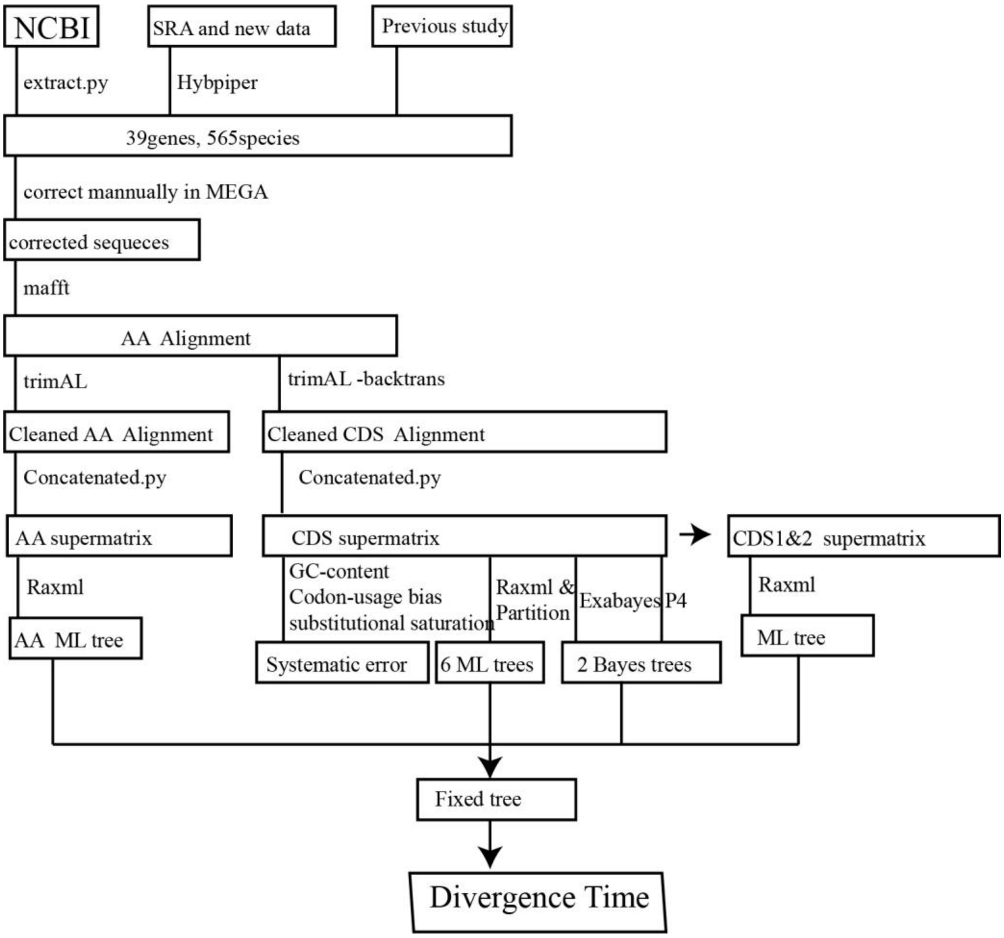

Supplemental Figure 1 Main workflow for phylogenetic reconstruction in this study

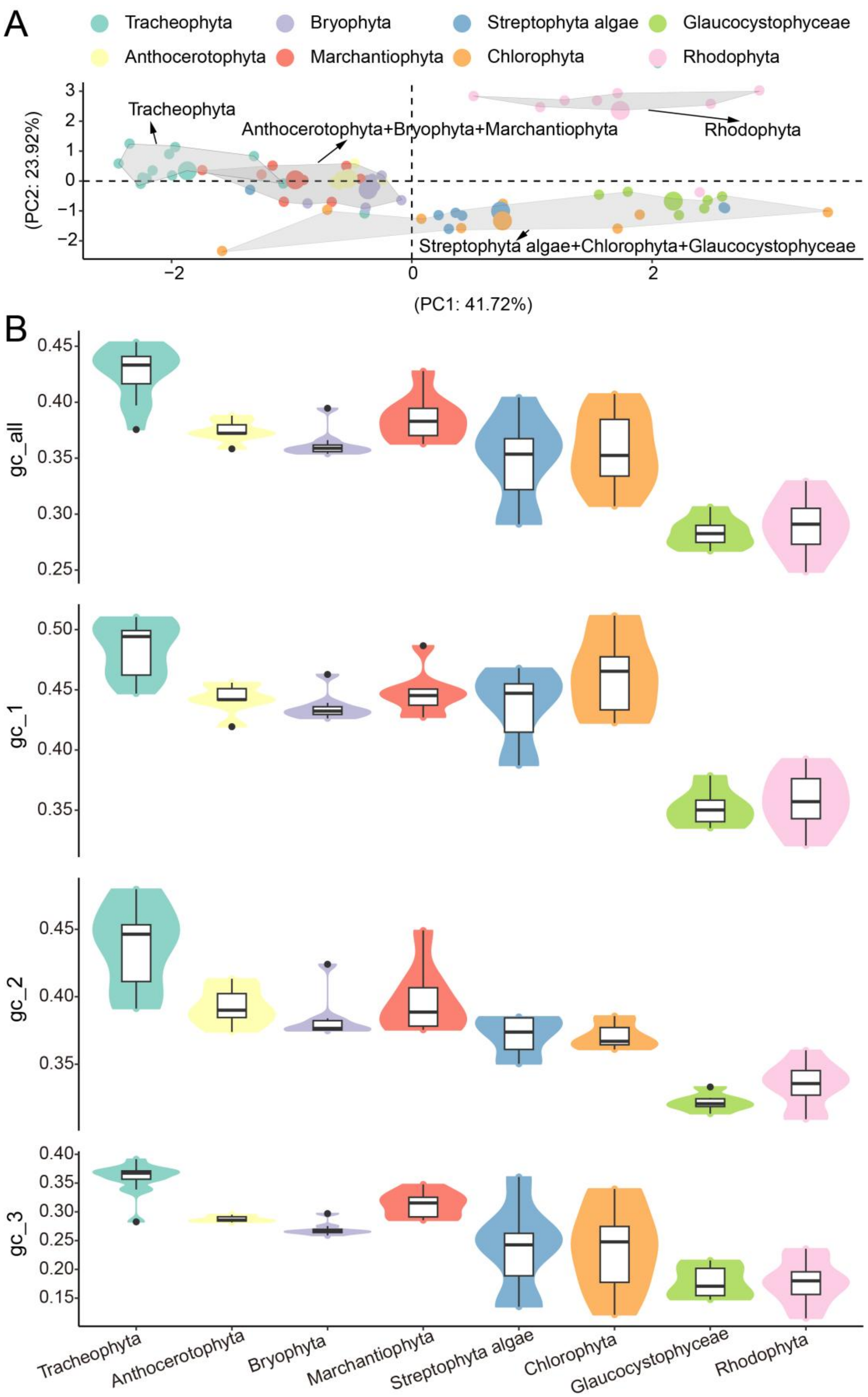

Supplemental Figure 2 Molecular features of protein coding DNA from 65 selected genomes with six Glaucocystophyceae species included.

(A) PCA analysis of codon-usage bias, all the clusters divided by either PC1 or PC2 are marked by grey shadows. **(B)** Violine plots showing GC-content differences, with the x-axis representing 8 main lineages in green plants and the outgroup Rhodophyta. Different lineages are marked in different colors.

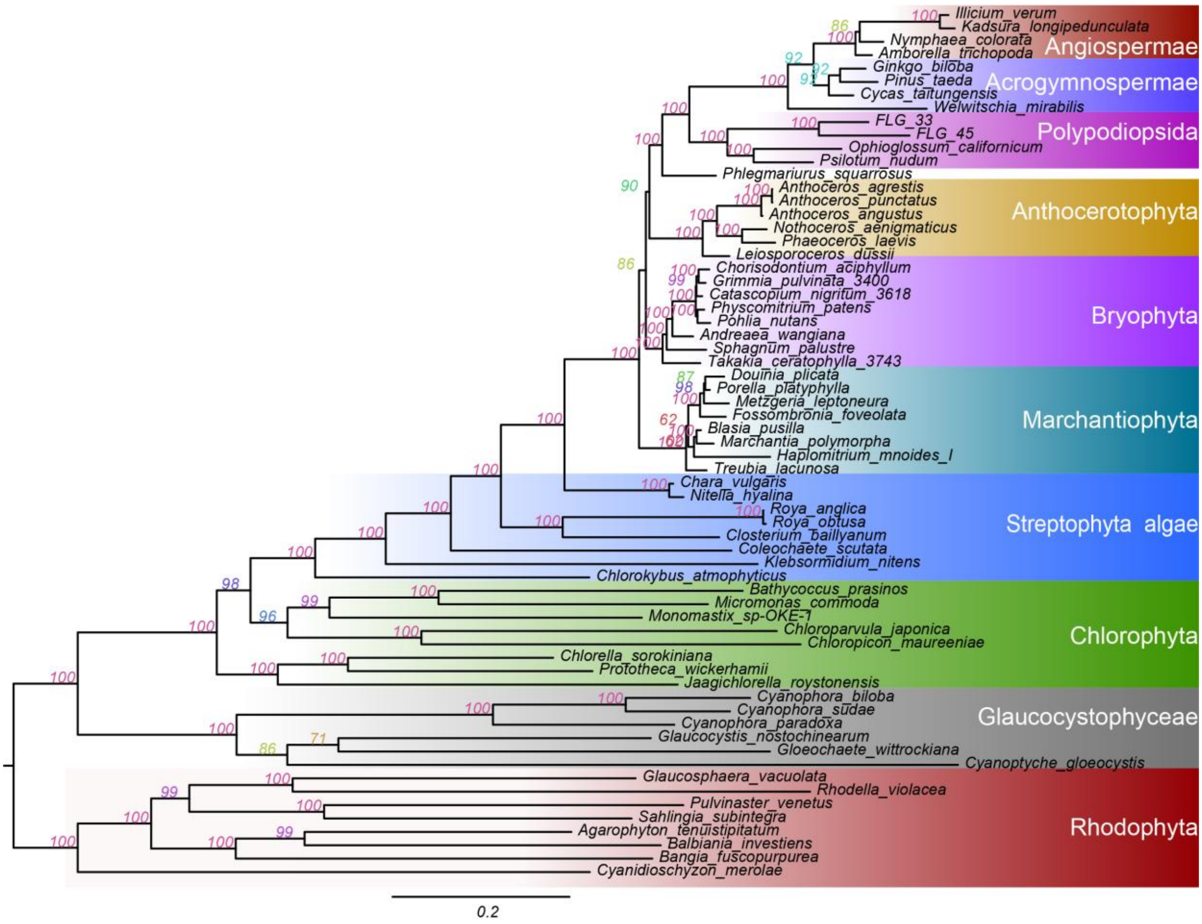

Supplemental Figure 3 Maximum likelihood tree inferred by protein coding genes for 65-species data.

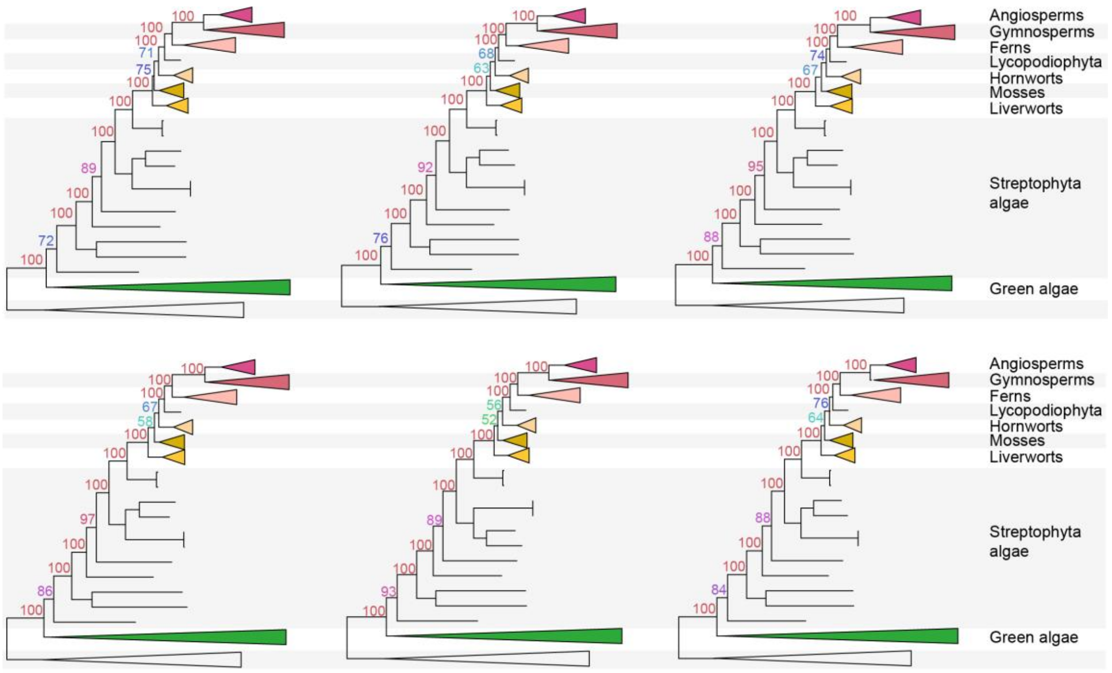

Supplemental Figure 4 The phylogenetic results inferred from 6 partition schemes. From top left to bottom right: 1 partition, 2 partitions (the first and second codon positions for all genes as one partition, the others as one partition), 3 partitions (each codon position for all genes as one partition), 39 partitions (each gene locus as one partition), 78 partitions (the first and second codon positions for each gene together as one partition, the third codon position for each gene as one partition), 117 partitions (each codon position in each gene locus as one partition). Bootstrap values for each backbone tree were marked on each node.

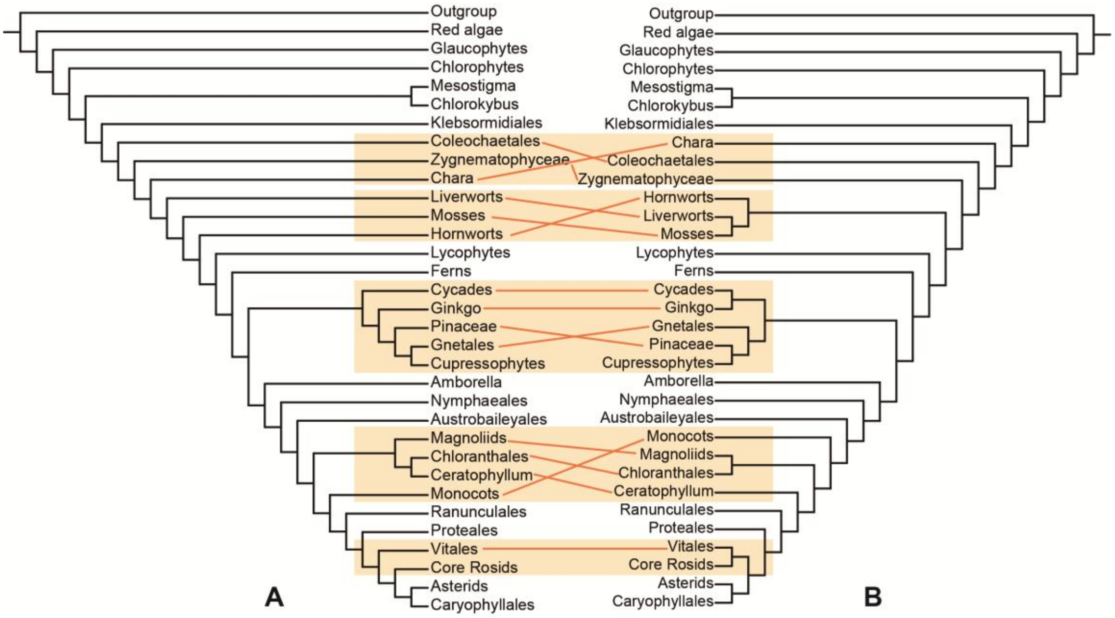

Supplemental Figure 5 Topological comparison of phylogeny between this study (A) and 1KP paper(B)

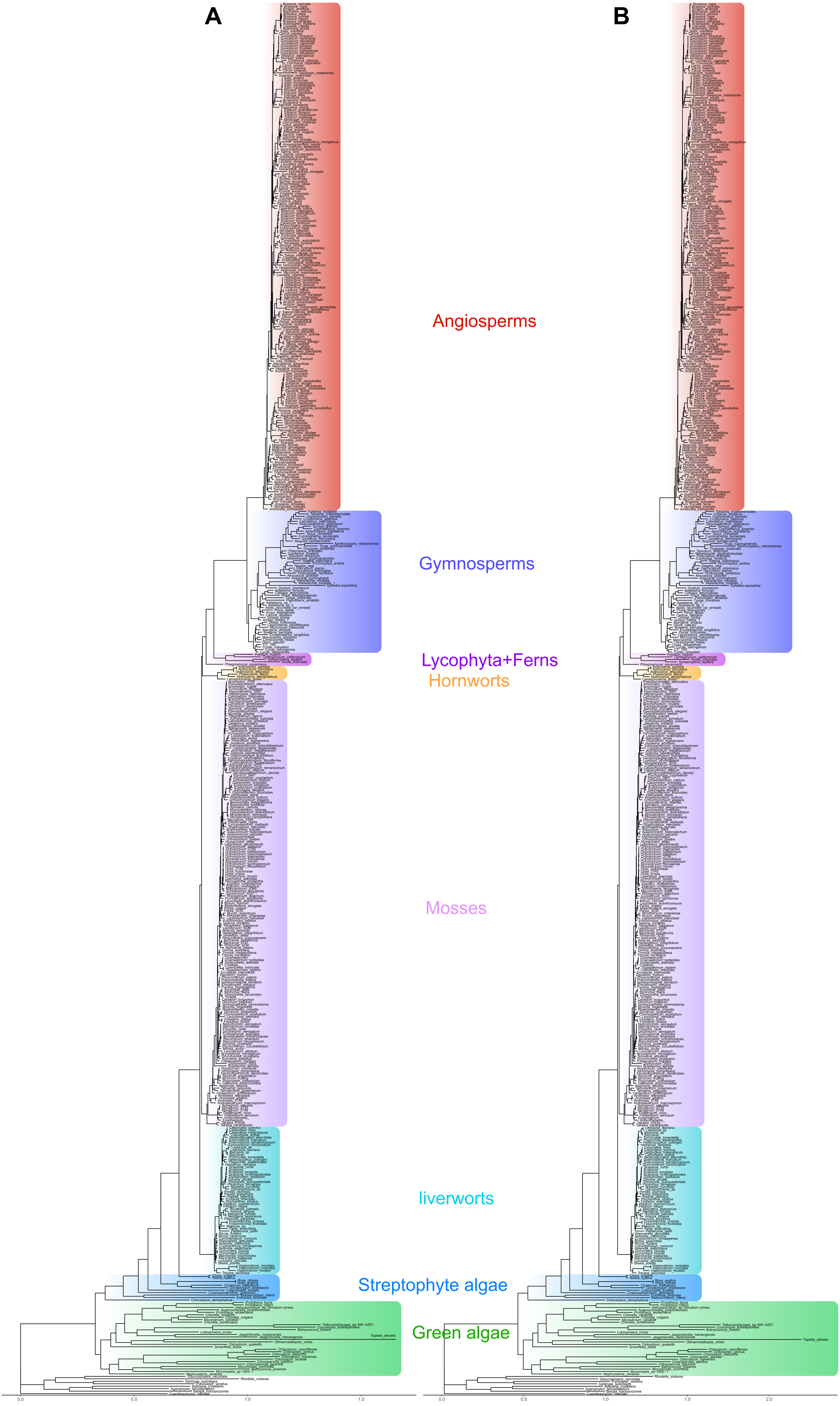

Supplemental Figure 6 The phylogenetic results of partition scheme 1 and 2

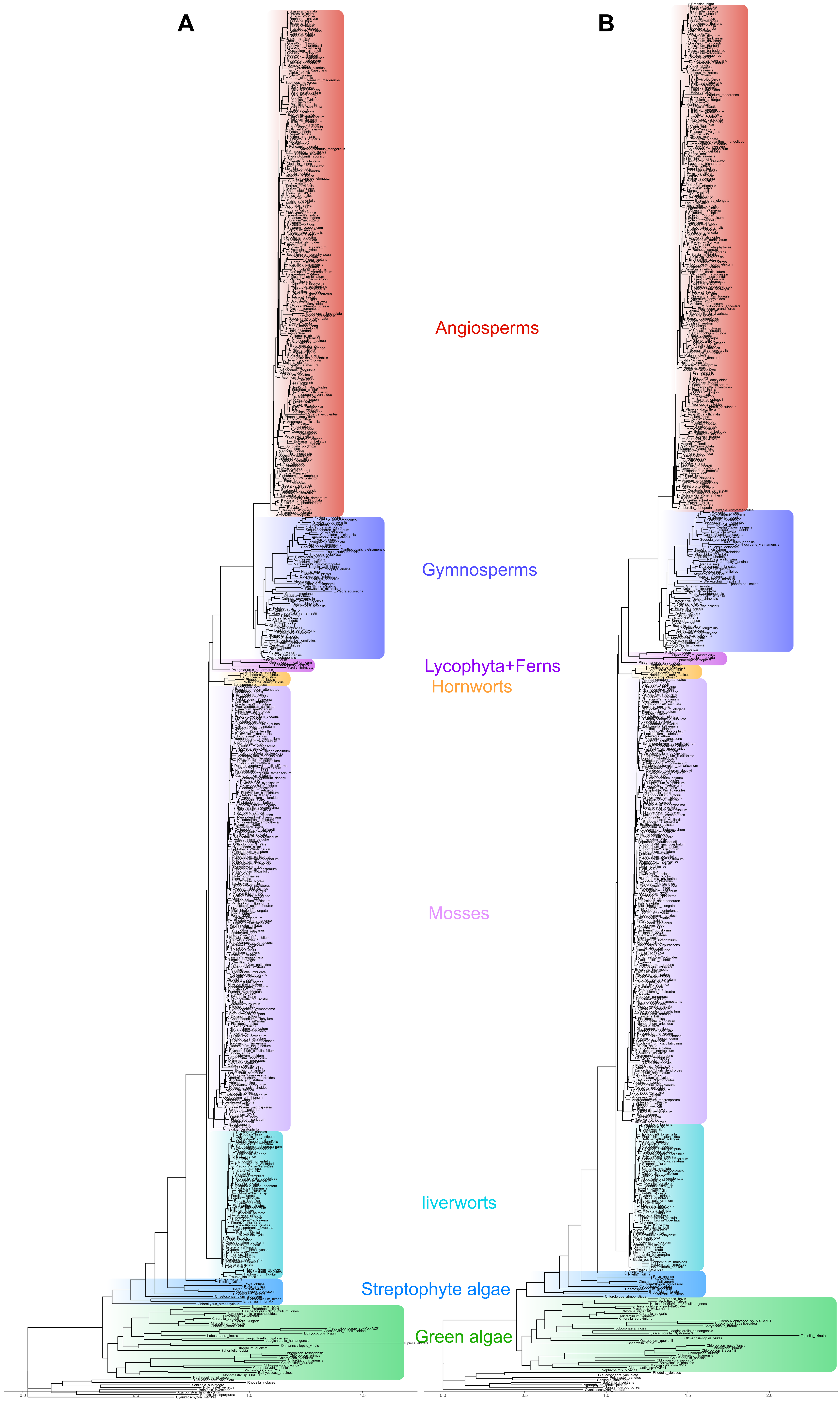

Supplemental Figure 7 The phylogenetic results of partition scheme 3 and 4

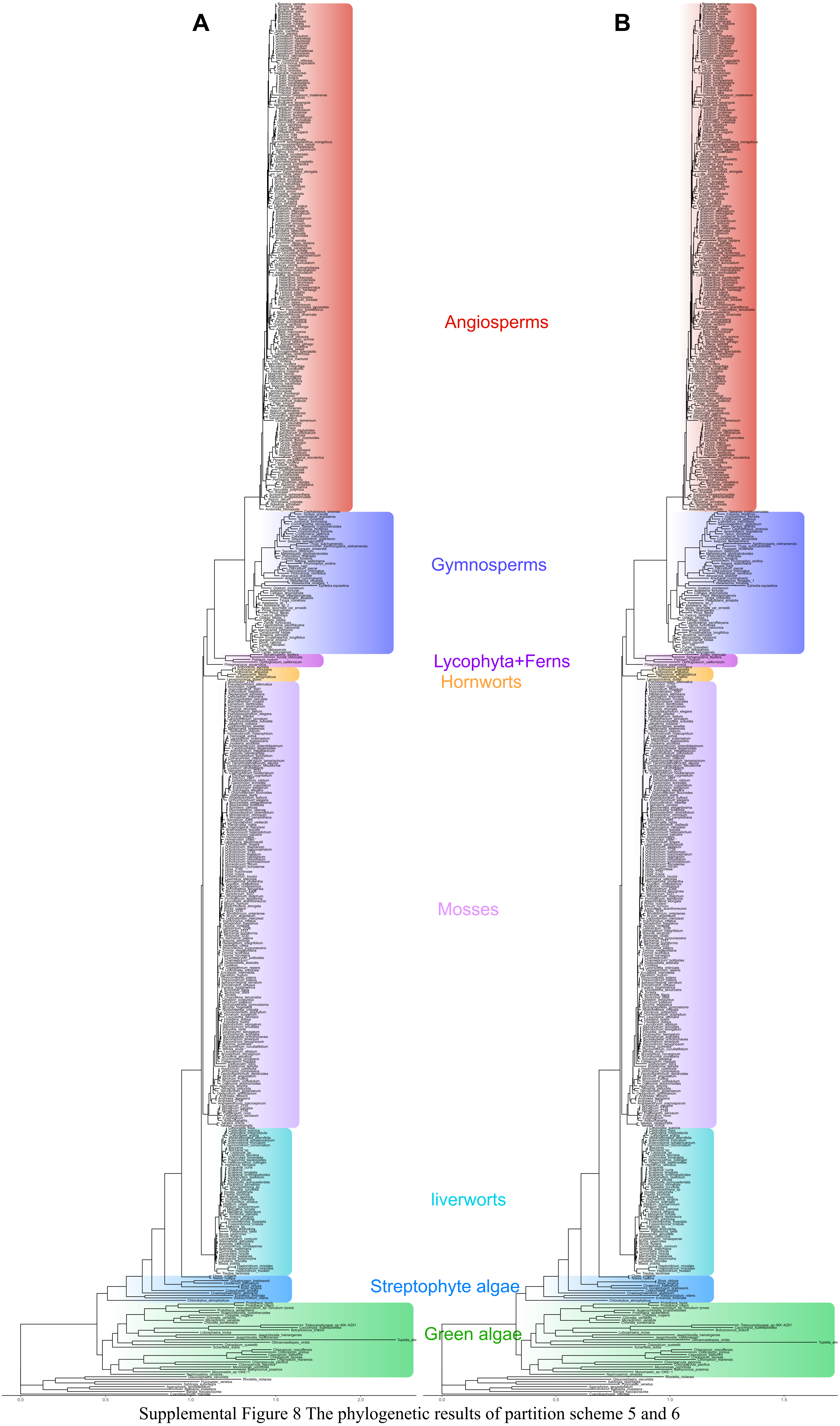

Supplemental Figure 8 The phylogenetic results of partition scheme 5 and 6

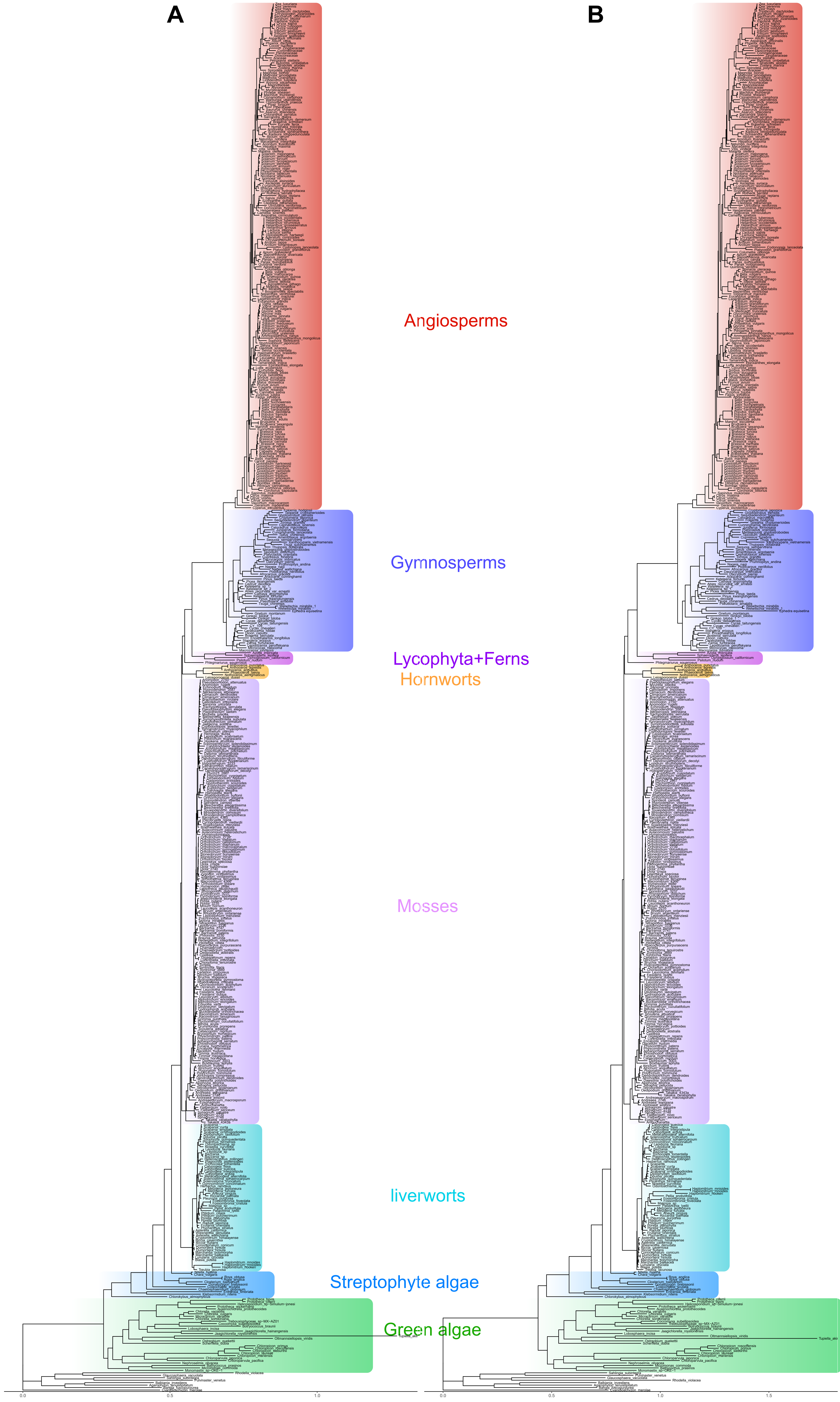

Supplemental Figure 9 The phylogenetic results of 1st+2nd codon positions and aa

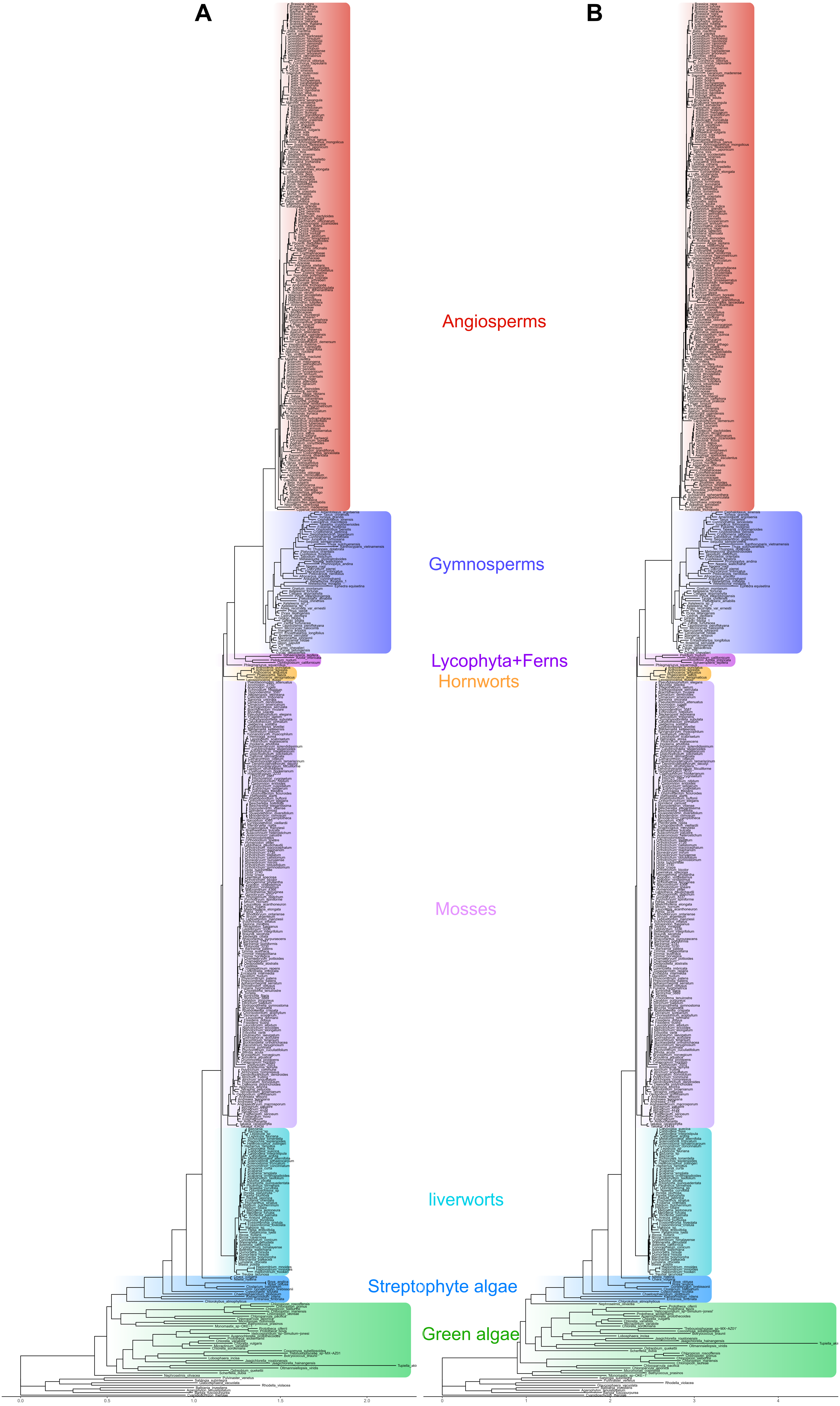

Supplemental Figure 10 The phylogenetic results of Exabayes and p4

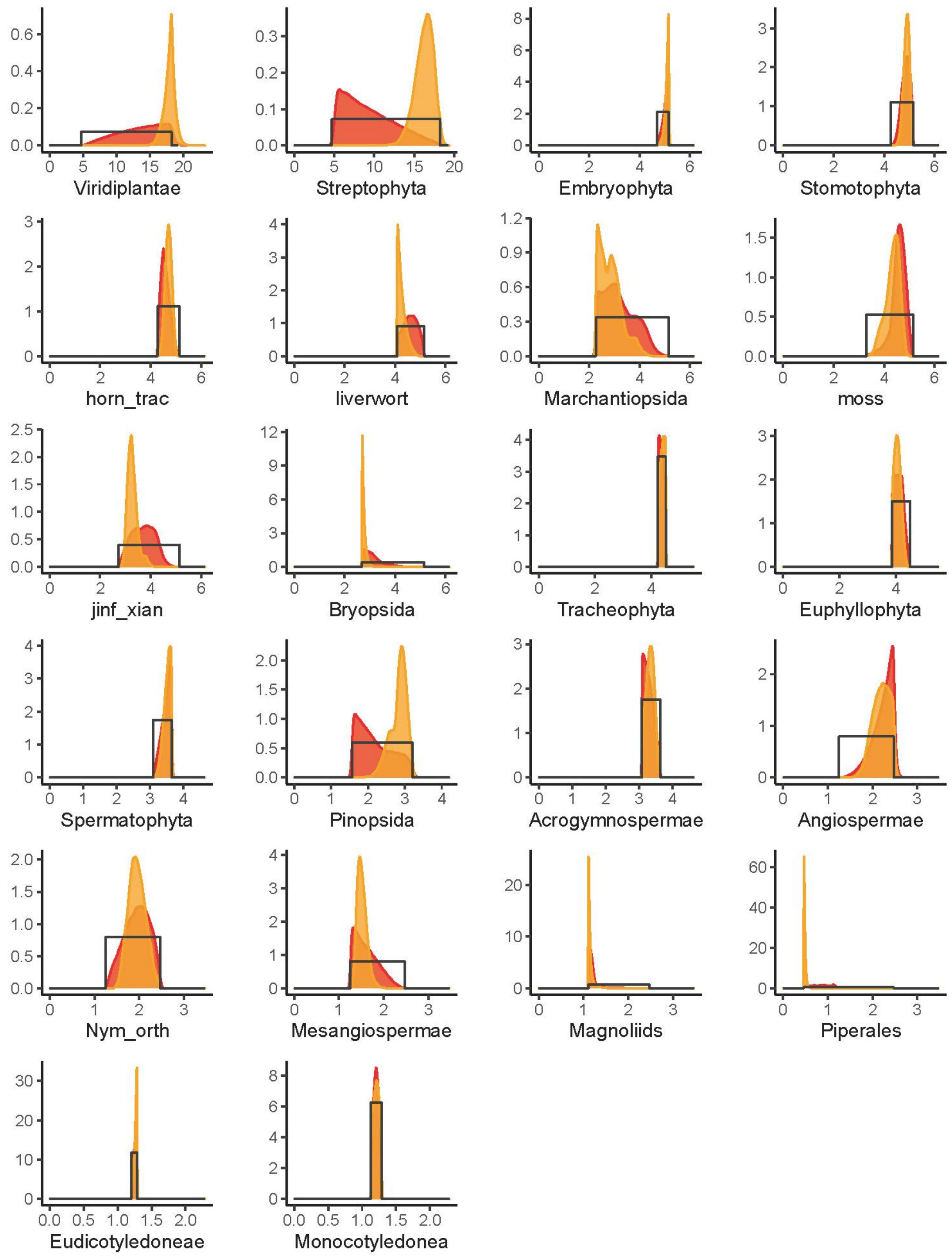

Supplemental Figure 11 Comparison of the age distributions on the 22 calibrated nodes for the specified priors (black line), effective priors (orange), and posteriors (red) on Strategy A1. The time unit for x axis is 100Ma.

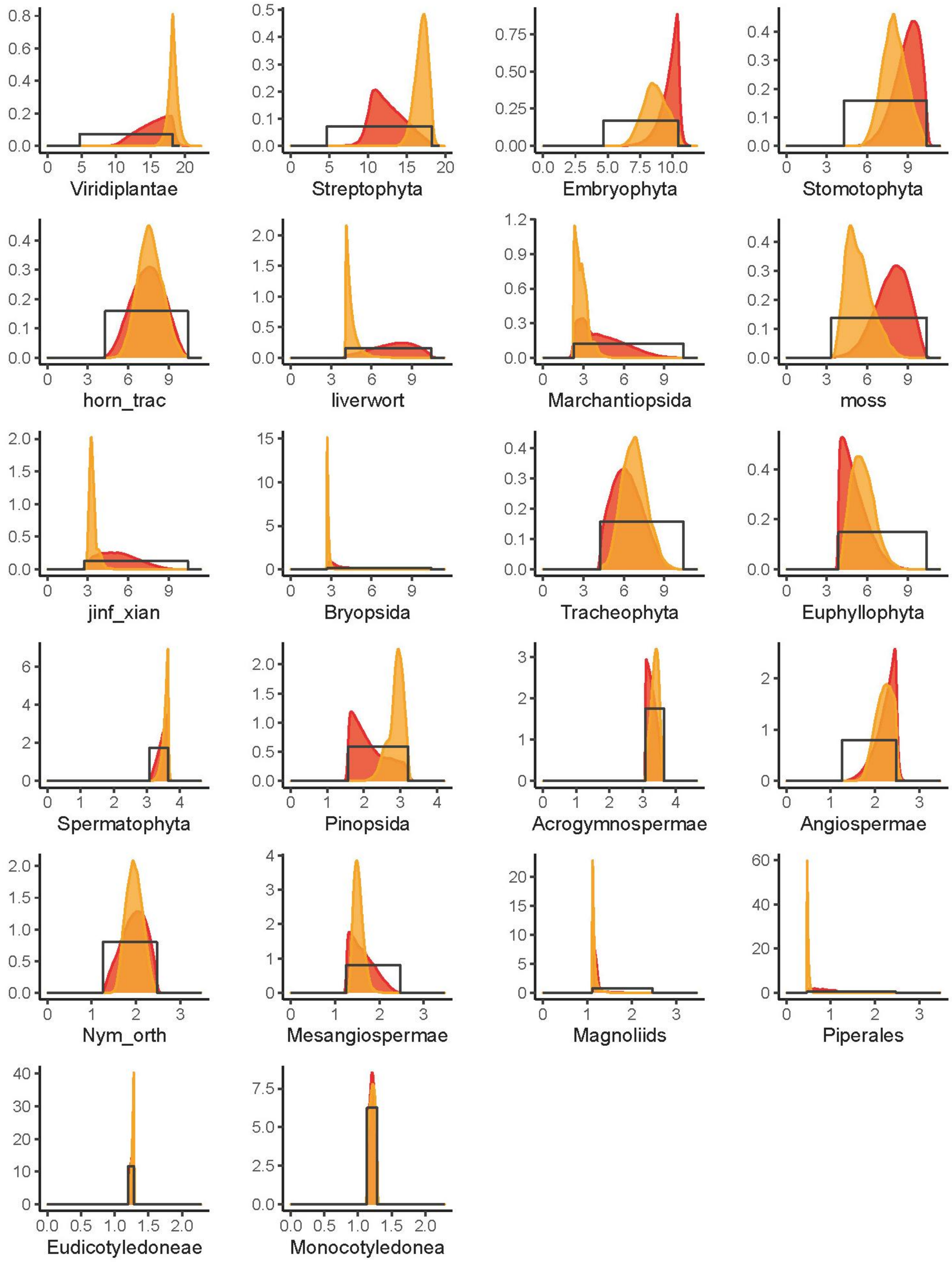

Supplemental Figure 12 Comparison of the age distributions on the 22 calibrated nodes for the specified priors (black line), effective priors (orange), and posteriors (red) on Strategy A2. The time unit for x axis is 100Ma.

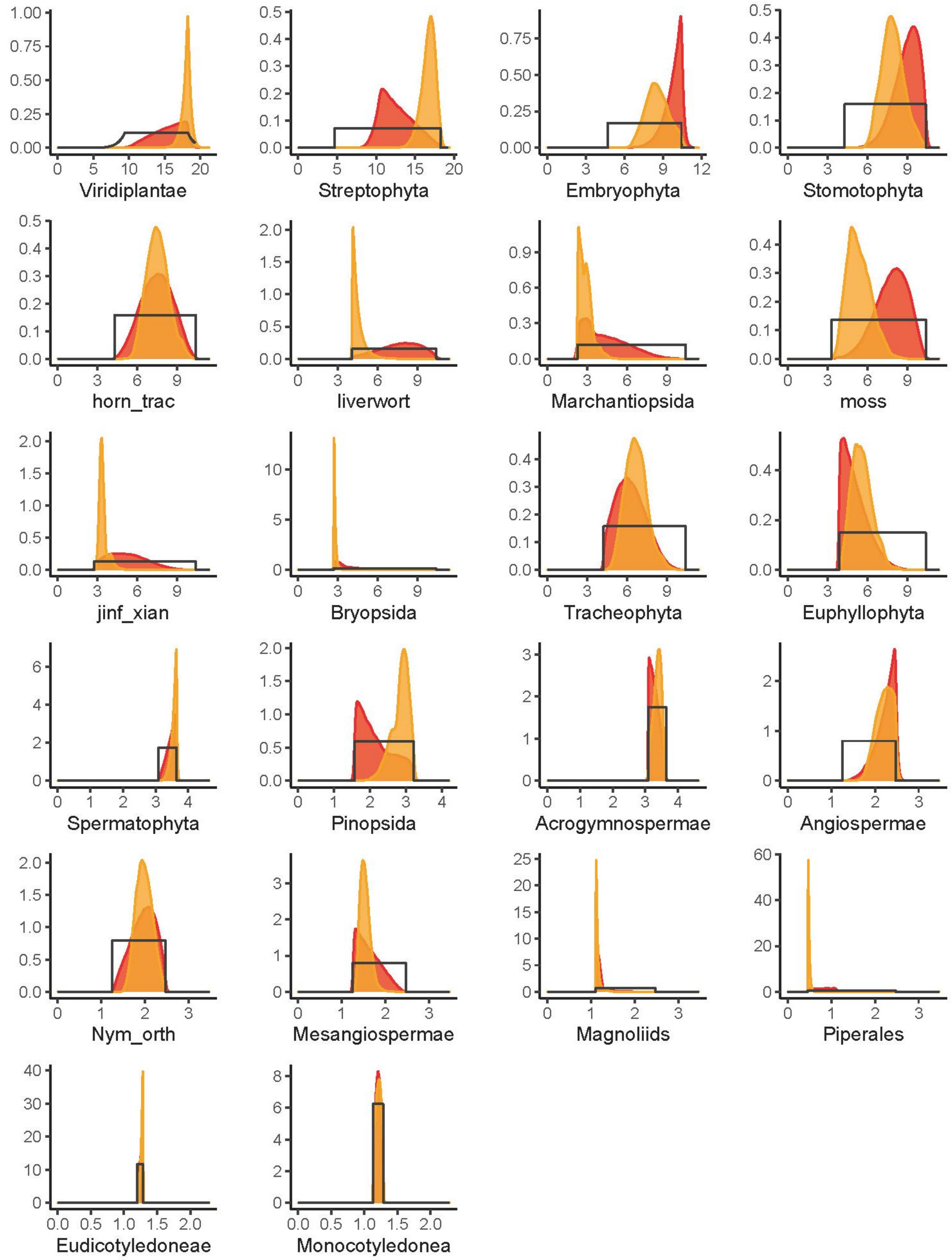

Supplemental Figure 13 Comparison of the age distributions on the 22 calibrated nodes for the specified priors (black line), effective priors (orange), and posteriors (red) on Strategy A3. The time unit for x axis is 100Ma.

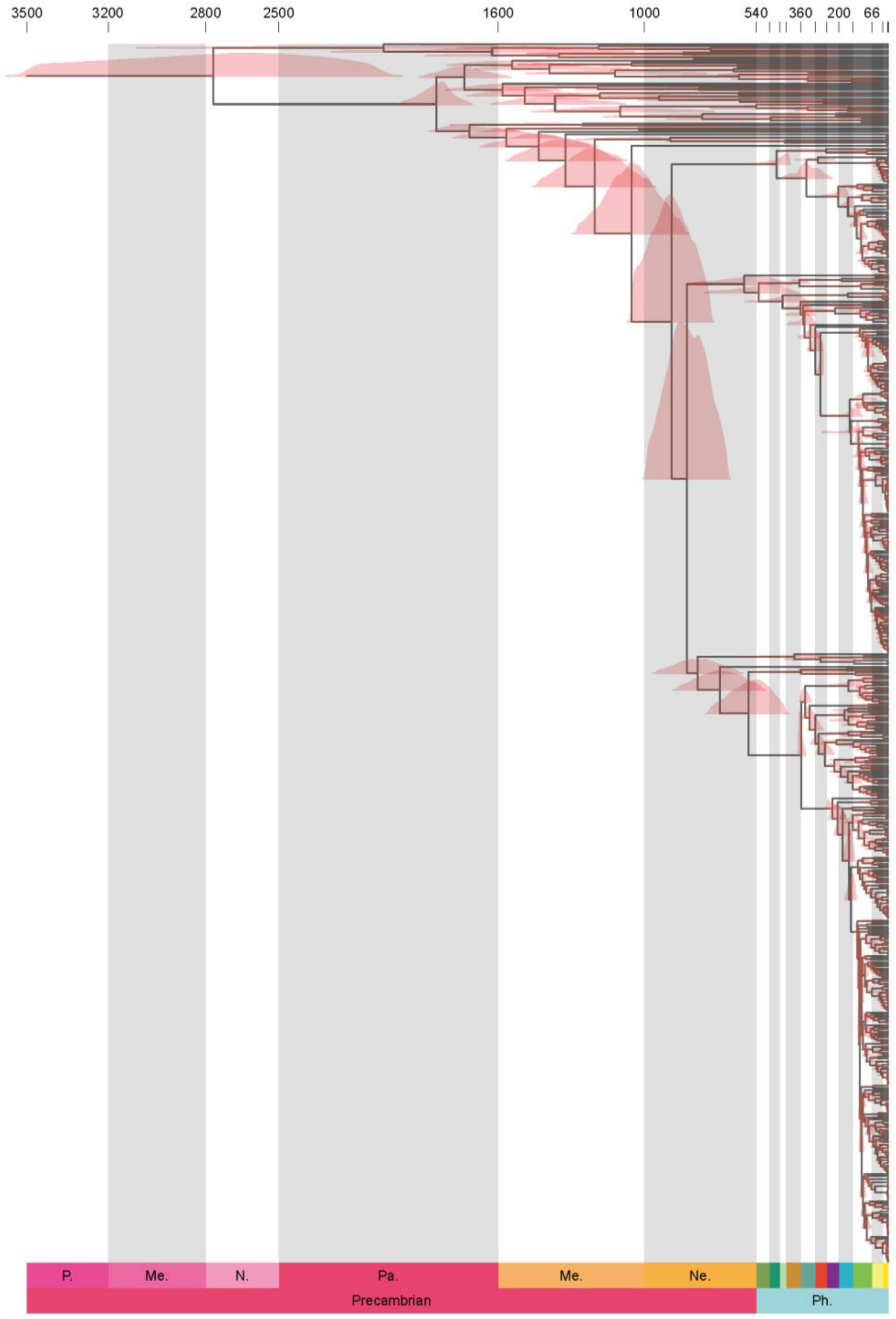

Supplemental Figure 14 Time tree estimated from strategy B1

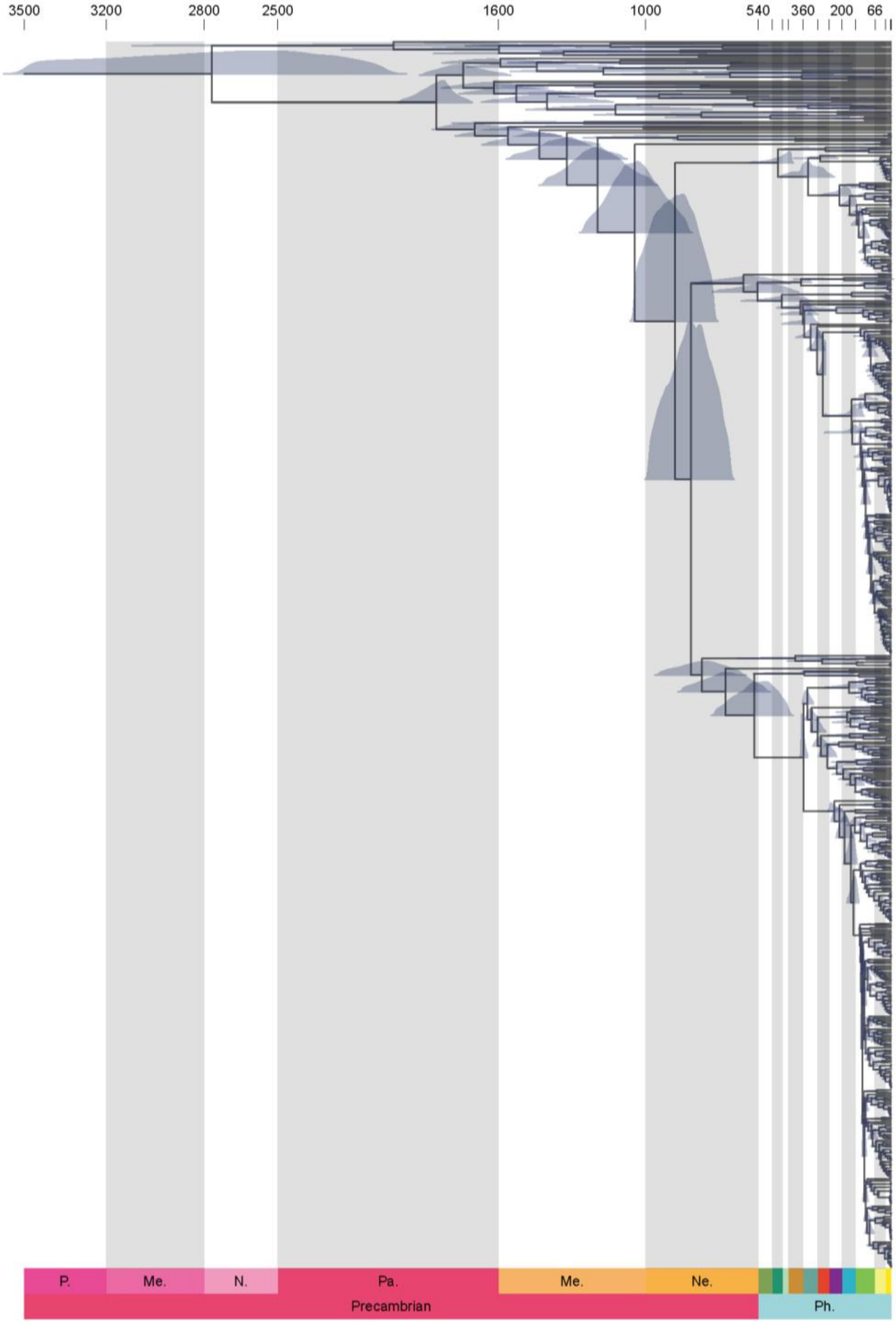

Supplemental Figure 15 Time tree estimated from strategy B2

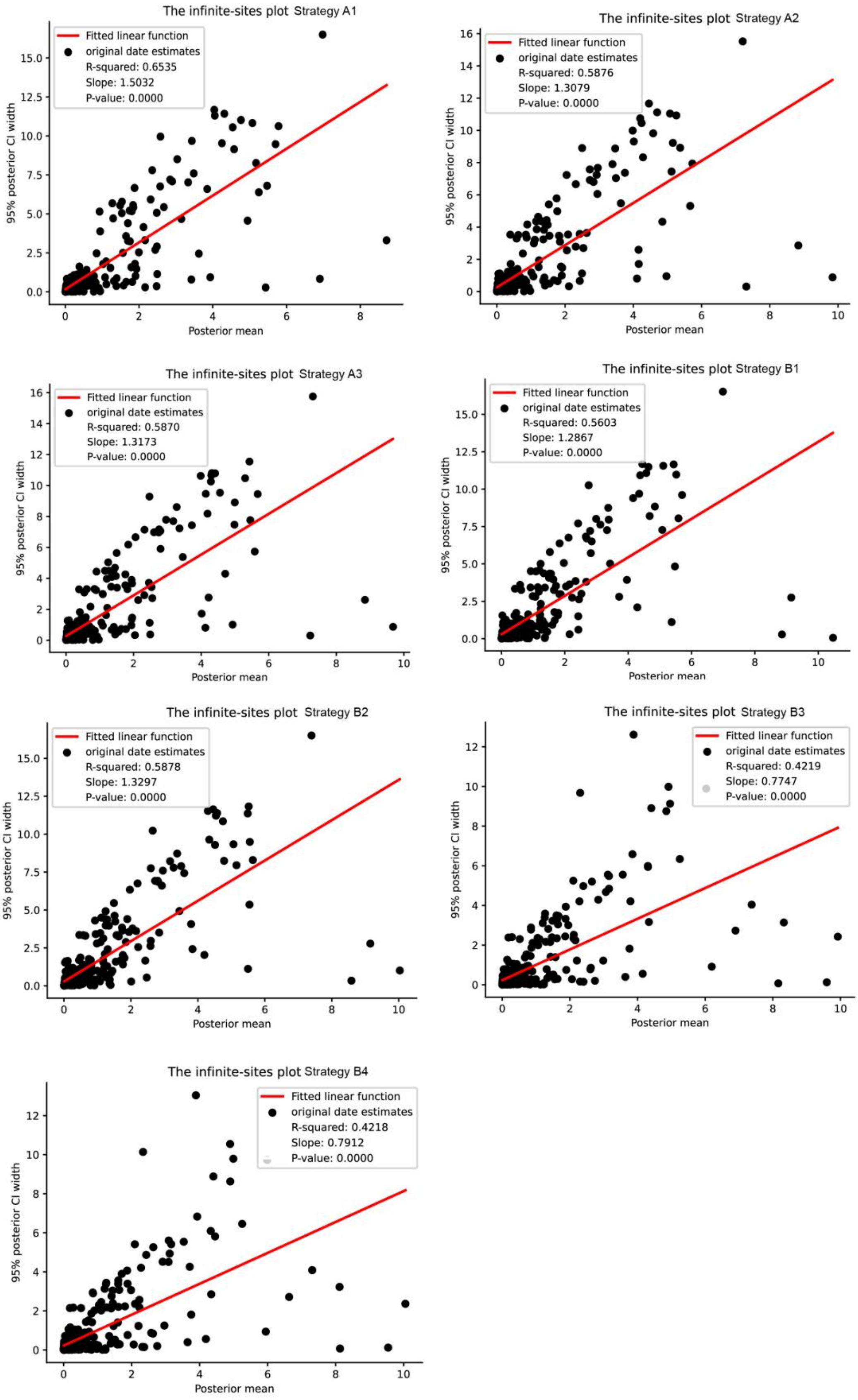

The infinite-sites plot Strategy A1
Fitted linear function
original date estimates
R-squared: 0.6535
Slope: 1.5032
P-value: 0.0000
95% posterior CI width
Posterior mean
The infinite-sites plot Strategy A2
Fitted linear function
original date estimates
R-squared: 0.5876
Slope: 1.3079
P-value: 0.0000
95% posterior CI width
Posterior mean
The infinite-sites plot Strategy A3
Fitted linear function
original date estimates
R-squared: 0.5870
Slope: 1.3173
P-value: 0.0000
95% posterior CI width
Posterior mean
The infinite-sites plot Strategy B1
Fitted linear function
original date estimates
R-squared: 0.5603
Slope: 1.2867
P-value: 0.0000
95% posterior CI width
Posterior mean
The infinite-sites plot Strategy B2
Fitted linear function
original date estimates
R-squared: 0.5878
Slope: 1.3297
P-value: 0.0000
95% posterior CI width
Posterior mean
The infinite-sites plot Strategy B3
Fitted linear function
original date estimates
R-squared: 0.4219
Slope: 0.7747
P-value: 0.0000
95% posterior CI width
Posterior mean
The infinite-sites plot Strategy B4
Fitted linear function
original date estimates
R-squared: 0.4218
Slope: 0.7912
P-value: 0.0000
95% posterior CI width
Posterior mean

Supplemental Figure 16 The infinite site plots. Widths of the 95% posterior CIs plotted against the posterior means of divergence times for seven strategies. The low R^2 values indicate that some of the estimation errors can be attributed to the limited amount of sequence data. The regression coefficient (slope) reflects the degree of precision in the fossil calibrations.

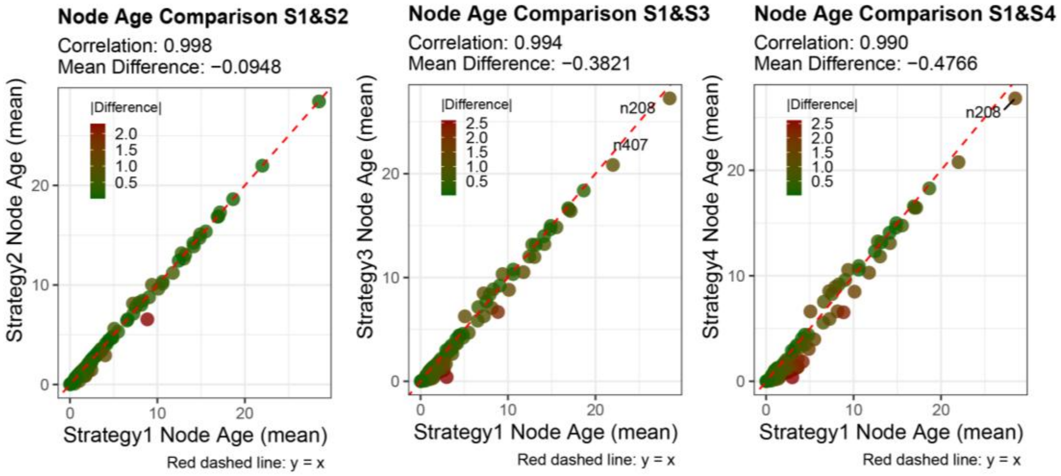

Supplemental Figure 17 Comparison of divergence-time estimates for 207 taxa, based on different sets of fossil calibrations.
